# Mortality in Iraq Associated with the 2003–2011 War and Occupation: Findings from a National Cluster Sample Survey by the University Collaborative Iraq Mortality Study

**DOI:** 10.1371/journal.pmed.1001533

**Published:** 2013-10-15

**Authors:** Amy Hagopian, Abraham D. Flaxman, Tim K. Takaro, Sahar A. Esa Al Shatari, Julie Rajaratnam, Stan Becker, Alison Levin-Rector, Lindsay Galway, Berq J. Hadi Al-Yasseri, William M. Weiss, Christopher J. Murray, Gilbert Burnham

**Affiliations:** 1 Health Alliance International, Department of Global Health, University of Washington, Seattle, Washington, United States of America; 2 Institute for Health Metrics and Evaluation, Department of Global Health, University of Washington, Seattle, Washington, United States of America; 3 Simon Fraser University, Burnaby, British Columbia, Canada; 4 Human Resources Development and Training Center, Iraq Ministry of Health, Baghdad, Iraq; 5 Harborview Medical Center, Department of Global Health, University of Washington, Seattle, Washington, United States of America; 6 Johns Hopkins University, Baltimore, Maryland, United States of America; 7 Iraq Ministry of Health, Baghdad, Iraq; University of Ottawa, Canada

## Abstract

**Background:**

Previous estimates of mortality in Iraq attributable to the 2003 invasion have been heterogeneous and controversial, and none were produced after 2006. The purpose of this research was to estimate direct and indirect deaths attributable to the war in Iraq between 2003 and 2011.

**Methods and Findings:**

We conducted a survey of 2,000 randomly selected households throughout Iraq, using a two-stage cluster sampling method to ensure the sample of households was nationally representative. We asked every household head about births and deaths since 2001, and all household adults about mortality among their siblings. We used secondary data sources to correct for out-migration. From March 1, 2003, to June 30, 2011, the crude death rate in Iraq was 4.55 per 1,000 person-years (95% uncertainty interval 3.74–5.27), more than 0.5 times higher than the death rate during the 26-mo period preceding the war, resulting in approximately 405,000 (95% uncertainty interval 48,000–751,000) excess deaths attributable to the conflict. Among adults, the risk of death rose 0.7 times higher for women and 2.9 times higher for men between the pre-war period (January 1, 2001, to February 28, 2003) and the peak of the war (2005–2006). We estimate that more than 60% of excess deaths were directly attributable to violence, with the rest associated with the collapse of infrastructure and other indirect, but war-related, causes. We used secondary sources to estimate rates of death among emigrants. Those estimates suggest we missed at least 55,000 deaths that would have been reported by households had the households remained behind in Iraq, but which instead had migrated away. Only 24 households refused to participate in the study. An additional five households were not interviewed because of hostile or threatening behavior, for a 98.55% response rate. The reliance on outdated census data and the long recall period required of participants are limitations of our study.

**Conclusions:**

Beyond expected rates, most mortality increases in Iraq can be attributed to direct violence, but about a third are attributable to indirect causes (such as from failures of health, sanitation, transportation, communication, and other systems). Approximately a half million deaths in Iraq could be attributable to the war.

*Please see later in the article for the Editors' Summary*

## Abstract 2

Based on a survey of 2,000 randomly selected households throughout Iraq, Amy Hagopian and colleagues estimate that close to half a million excess deaths are attributable to the recent Iraq war and occupation.

*Please see later in the article for the Editors' Summary*

## Editors' Summary

**Background:**

War is a major public health problem. Its health effects include violent deaths among soldiers and civilians as well as indirect increases in mortality and morbidity caused by conflict. Unlike those of other causes of death and disability, however, the consequences of war on population health are rarely studied scientifically. In conflict situations, deaths and diseases are not reliably measured and recorded, and estimating the proportion caused, directly or indirectly, by a war or conflict is challenging. Population-based mortality survey methods—asking representative survivors about deaths they know about—were developed by public health researchers to estimate death rates. By comparing death rate estimates for periods before and during a conflict, researchers can derive the number of excess deaths that are attributable to the conflict.

**Why Was This Study Done?:**

A number of earlier studies have estimated the death toll in Iraq since the beginning of the war in March 2003. The previous studies covered different periods from 2003 to 2006 and derived different rates of overall deaths and excess deaths attributable to the war and conflict. All of them have been controversial, and their methodologies have been criticized. For this study, based on a population-based mortality survey, the researchers modified and improved their methodology in response to critiques of earlier surveys. The study covers the period from the beginning of the war in March 2003 until June 2011, including a period of high violence from 2006 to 2008. It provides population-based estimates for excess deaths in the years after 2006 and covers most of the period of the war and subsequent occupation.

**What Did the Researchers Do and Find?:**

Interviewers trained by the researchers conducted the survey between May 2011 and July 2011 and collected data from 2,000 randomly selected households in 100 geographical clusters, distributed across Iraq's 18 governorates. The interviewers asked the head of each household about deaths among household members from 2001 to the time of the interview, including a pre-war period from January 2001 to March 2003 and the period of the war and occupation. They also asked all adults in the household about deaths among their siblings during the same period. From the first set of data, the researchers calculated the crude death rates (i.e., the number of deaths during a year per 1,000 individuals) before and during the war. They found the wartime crude death rate in Iraq to be 4.55 per 1,000, more than 50% higher than the death rate of 2.89 during the two-year period preceding the war. By multiplying those rates by the annual Iraq population, the authors estimate the total excess Iraqi deaths attributable to the war through mid-2011 to be about 405,000. The researchers also estimated that an additional 56,000 deaths were not counted due to migration. Including this number, their final estimate is that approximately half a million people died in Iraq as a result of the war and subsequent occupation from March 2003 to June 2011.

The risk of death at the peak of the conflict in 2006 almost tripled for men and rose by 70% for women. Respondents attributed 20% of household deaths to war-related violence. Violent deaths were attributed primarily to coalition forces (35%) and militia (32%). The majority (63%) of violent deaths were from gunshots. Twelve percent were attributed to car bombs. Based on the responses from adults in the surveyed households who reported on the alive-or-dead status of their siblings, the researchers estimated the total number of deaths among adults aged 15–60 years, from March 2003 to June 2011, to be approximately 376,000; 184,000 of these deaths were attributed to the conflict, and of those, the authors estimate that 132,000 were caused directly by war-related violence.

**What Do These Findings Mean?:**

These findings provide the most up-to-date estimates of the death toll of the Iraq war and subsequent conflict. However, given the difficult circumstances, the estimates are associated with substantial uncertainties. The researchers extrapolated from a small representative sample of households to estimate Iraq's national death toll. In addition, respondents were asked to recall events that occurred up to ten years prior, which can lead to inaccuracies. The researchers also had to rely on outdated census data (the last complete population census in Iraq dates back to 1987) for their overall population figures. Thus, to accompany their estimate of 460,000 excess deaths from March 2003 to mid-2011, the authors used statistical methods to determine the likely range of the true estimate. Based on the statistical methods, the researchers are 95% confident that the true number of excess deaths lies between 48,000 and 751,000—a large range. More than two years past the end of the period covered in this study, the conflict in Iraq is far from over and continues to cost lives at alarming rates. As discussed in an accompanying Perspective by Salman Rawaf, violence and lawlessness continue to the present day. In addition, post-war Iraq has limited capacity to re-establish and maintain its battered public health and safety infrastructure.

**Additional Information:**

Please access these websites via the online version of this summary at http://dx.doi.org/10.1371/journal.pmed.1001533

- This study is further discussed in a PLOS Medicine Perspective by Salman Rawaf.
- The Geneva Declaration on Armed Violence and Development website provides information on the global burden of armed violence.
- The International Committee of the Red Cross provides information about war and international humanitarian law (in several languages).
- Medact, a global health charity, has information on health and conflict.
- Columbia University has a program on forced migration and health.
- Johns Hopkins University runs the Center for Refugee and Disaster Response.
- University of Washington's Health Alliance International website also has information about war and conflict.

## Introduction

Estimates of the number of Iraqi deaths after the US-led invasion in 2003 have varied considerably and are contested [1]–[24]. Measuring deaths during war is complex, and methods vary, yet assessing the public health consequences of armed conflict is important [25].

There are several approaches to measuring mortality during a period of conflict, including registration of vital events, passive surveillance, and population-based surveys [26]. Iraq's last complete census was in 1987, with a partial census in 1997, after which Iraq experienced a period of extensive demographic change, including internal and external migration. With regard to vital events, death certificates continued to be issued during the conflict, though aggregation and tabulation were affected [27].

To date, five population-based surveys have attempted to estimate war-related deaths in Iraq. None of these were conducted after 2006, the peak of the conflict and subsequent migration [4]. Two of these studies reported only the violent death rate [28],[29], and three estimated both violent-only and all-cause death rates [30]–[32]. These studies reported widely varying rates of mortality. All attracted various criticisms, including potential bias in sample selection, wide ranges of uncertainty intervals (UIs) related to relatively small sample sizes, and disputes related to statistical methods, the choice of reference populations for calculating rates, and the plausibility of results [4].

There has been substantial demographic change in Iraq as a result of both internal and cross-border migration throughout the course of the long conflict. Our study builds on lessons from previous mortality studies in conflict settings and, to our knowledge, provides the first estimates for mortality in Iraq during the years 2006–2011. We used both the standard household demographic method (reported household deaths) and the improved corrected sibling survival (ICSS) method [33], the latter to increase sample size, correct for survival bias [33]–[35], and reduce migration bias. We analyzed data from both methods to produce nationally representative estimates of conflict-related mortality for both the general population (household survey) and for Iraqi adults, defined [36] as those aged 15–60 y (sibling survey). Additional analyses of secondary data were performed to adjust these estimates to account for migration.

## Methods

In mid-2011 we conducted a nationally representative cross-sectional survey of all adults living in 2,000 randomly selected households in 100 clusters across Iraq. In retrospective cluster sample mortality surveys, the idea is to use random selection to generate a predetermined number of “clusters,” or geographically proximate household groups, across the area in question. These representative households are then queried about their composition and mortality events over a given time period, to allow researchers to generate crude death rates; these rates are then multiplied by the country's population total to calculate a death estimate [37]. We used a questionnaire that asked all adults in the household about the births and deaths of their siblings, as well as all births and deaths in the household since 2001. The questionnaire is provided as Questionnaire S1.

### Setting and Sample Selection

We employed a two-stage cluster sampling method. We used a commercial software product (LandScan) that contained gridded population data at the 1-km^2^ level in a geographic information system, and we linked it to Google Earth imagery. In the first stage of cluster selection, we randomly selected 100 1-km^2^ areas using a probability-proportional-to-size approach. After those areas were selected, we superimposed a smaller grid (10 m×10 m) onto each of the selected areas, and randomly selected one grid cell in each of the 100 clusters. In each small grid cell, we examined the Google Earth image and selected the residential rooftop that most fully fit in the square to serve as the start household [38]. Details are in Figure S1, Text S2, and Questionnaire S1. Our field manual (see Manual S1) established protocols for selecting 19 dwellings adjacent to the starting household.

Our sample size was established building on experience derived from previous studies. By doubling the number of clusters used in two previous mortality studies [30],[31], we were able to reduce the possibility of missing pockets of unusually high or low conflict-related mortality, and by halving the number of households per cluster we were able to keep the operational complexity of conducting the survey manageable, and still visit a reasonable number of households per cluster.

### Processes and Timeline

We recruited study collaborators and drafted questionnaires in early 2011. Lead researchers from three North American universities and two Iraqi team leaders met in northern Iraq in March 2011 to revise data collection instruments and survey processes, finalize the field manual, and gain experience finding start households using Google maps. The two Iraqi team leaders recruited eight medical doctors with experience in community surveys as interviewers. Author W. M. W. conducted training for data collectors in Iraq in March 2011. Weekly (or more frequent) teleconferences were held between the North American team and the lead Iraqi investigator during the design and implementation phases. The entire team met again in Iraq in September 2011 to review and interpret preliminary findings.

### Data Collection and Entry

Four two-person teams along with their supervisors (for a total of ten surveyors) surveyed 100 clusters of 20 households between May 13 and July 2, 2011. The supervisor returned to one randomly selected household in each cluster (where he or she had not previously visited) to repeat the survey as a quality check. When in governorates outside Baghdad, persons familiar with locations and local security issues joined the teams to help obtain local approvals and find designated clusters.

An Iraqi events calendar and an age/birth-year chart were created to assist with recalling dates of birth or death. Interviewers asked for causes of death, and coded these from a brief listing of common causes. For war-related deaths, we asked for specific causes (such as gunshots or explosions) and perceived responsible parties (such as coalition forces or criminals). We trained interviewers to probe for sensitive information about missing or disappeared persons, and about events distant in time among siblings and household members. We compiled qualitative observations about the remoteness and other characteristics of each cluster.

We needed to replace only one cluster (in Kerbela; governorate names per the Iraqi Central Statistical Organization [COSIT]; http://www.cosit.gov.iq/AAS/AAS2012/section_10/1.htm) for security reasons. We were obliged, however, to drop two remote clusters where our teams were strongly advised by community leaders not to visit dwellings (for cultural reasons); instead, household members were invited to a central location for interviews. As this violated the study protocol, these households were dropped from the analysis.

### Instruments

Each paper questionnaire contained a household and a sibling component. After obtaining verbal consent following our human subjects protocol, we interviewed the head of household (or the most senior member present) to complete a household listing. A household was defined as a group of people, not necessarily related, who regularly eat and sleep together in a building with a separate entrance and who share a kitchen. For the household component, household births and deaths between January 1, 2001, and the interview date were recorded. When deaths were reported, interviewers requested to see death certificates. We recorded whether interviewers were shown the certificate, whether the certificate was reported to be present but not seen, or whether it was absent.

The second component of the questionnaire was a sibling history module (commonly used in Demographic and Health Surveys in developing countries) [33]. By asking all adults in the household to recall and report on each of their siblings (defined as persons born to the same biological mother), we were able to estimate probabilities of death for adults across several decades.

Respondents to the sibling history module included all household members aged 18 y and older, and any married people under 18 y. Where necessary, telephone interviews of absent adult household members were conducted while the interviewers were in the household. We allowed limited proxy reporting for siblings. The mother of adult siblings in the home was allowed to report about her own children. If an adult in the home was incapable of responding (because of absence, disability, or refusal), his or her relatives reported on that person's siblings, but only if they said they were fully knowledgeable. Otherwise, the person's response was marked as missing. If two or more siblings lived together in the same home, we interviewed only one (whichever one was actually present, or, if all were present, the sibling with the nearest next birthday to the date of our visit).

Data were recorded on paper forms, and then entered using EpiData soon after collection (see full dataset at http://ghdx.healthmetricsandevaluation.org/record/mortality-iraq-associated-2003-2011-invasion-and-occupation). Data were immediately uploaded to a website “dashboard” to allow all investigators to monitor data collection. We employed algorithms to scan for systematic interviewer error [39], and observed none. All data records were rechecked against the paper record to identify and correct discrepancies.

### Analysis

#### Household analysis

We estimated crude death rates for the time periods January 1, 2001–February 28, 2003 and March 1, 2003–June 30, 2011 by counting deaths occurring in all households in each time period and dividing by the person-years lived within the time period. We collected month and year of birth and death information and month and year of household formation. When the value was missing for month of death (7%, *n* = 26), we used June (except for 2011, where we used March for the one case with the month missing).

We calculated UIs at the 95% level for crude death rates for each time period using a bootstrapping method. Uncertainty intervals can be interpreted similarly to confidence intervals. To account for clustering, we first sampled (with replacement) the 98 existing clusters 1,000 times, so that each time we selected 98 clusters—with some of the original clusters sampled more than once, and some not sampled at all. Next, for each of the 1,000 sets of clusters, we resampled the original number of households (with replacement) within each of the 98 sampled clusters. For each of these 1,000 replicates, we calculated annual crude death rates. The 2.5th and 97.5th percentiles of these 1,000 values served as our lower and upper bounds, respectively [40],[41].

To estimate excess deaths caused by conflict, we calculated the war-related death rate to be the difference between the crude death rate for each time period and the crude rate during the baseline time period (January 1, 2001, to February 28, 2003). To create a war-related death count for the total population, we used the yearly United Nations Population Division estimates [42] for Iraq multiplied by the war-related crude death rate. To estimate upper and lower uncertainty bounds, we used the bootstrapping method described above. Because the bootstrap process randomly chooses 1,000 possible scenarios, and we did not limit the assumptions otherwise, the occasional random selection could (and did) show a protective effect of conflict (which served to lower our final death rates).

To assess the effects of clustering on our data (“design effects”), we compared our two-stage bootstrap estimates of crude mortality to a naïve bootstrap: the ratio of the confidence interval of the larger to the smaller constitutes an estimate of the square root of the design effect. These effects of cluster sampling were not particularly large, ranging from 1.19 to 1.54 for each sex by year [43].

#### Sibling analysis

Data about adult mortality using the sibling report method are subject to predictable biases. Sibships that experience a higher mortality risk are underrepresented at the time of the survey, because these siblings are less likely to survive to be able to report (survival bias). Additionally, larger sibships are overrepresented in the sample, because there are more siblings in the sampling frame. We used the ICSS method to adjust for these biases [33]. Further details are in Text S1.

We calculated mortality rates for 5-y age groups between the ages of 15 and 59 y for the time periods January 1, 2001–February 28, 2003; March 1, 2003–December 31, 2004; the full years 2005–2006, 2007–2008, 2009–2010; and January 1–June 30, 2011. Our summary metric of adult mortality is _45_ *q*_15_, which is the risk that an individual will die before his or her 60th birthday given that he or she has lived to age 15 y. For example, male _45_ *q*_15_ ranges from below 0.05 in a few countries to above 0.45 in a handful of high-mortality African nations [44]. Uncertainty intervals were calculated using the same bootstrapping method as in the household analysis. Bootstrapping is appropriate for complex methods such as ICSS, where there is no alternative to calculating UIs. We used Stata/IC 12.0 and Python 2.6 for all analyses.

### Migration Adjustment

Unlike the adult sibling survival method, there is no accepted method for adjusting household figures to account for households entirely destroyed subsequent to the death of all members, or lost to migration out of the country, especially for households that experienced a death. The Iraq Family Health Survey (IFHS) study acknowledges this shortcoming in its work as well [32]. There is evidence that the killings in Iraq were disproportionately targeted towards the higher-income intelligentsia, a group typically in a better position to migrate to a safer setting if under attack [45]. We therefore reviewed a number of secondary data sources to estimate the number of Iraqis who migrated out of the country over the course of the war, to arrive at a total estimate of the missing households that left the country (and were therefore no longer available in our sampling frame). We then divided this total by an estimated household size, and multiplied total households by the average fraction of deaths per household [46] to estimate the total deaths our household survey would have missed, and added this number to our total death count.

### Ethical Review

We had review board approval from each participating institution in the study. Methods were reviewed to ensure they complied with the ethical guidelines for epidemiological research set out by the Council for International Organizations of Medical Sciences and other guidance, including the professional responsibility code of the American Association for Public Opinion Research [47]–[49]. An ethicist experienced in international research associated with the Institute of Translational Health Sciences at the University of Washington, Benjamin Wilfond, further reviewed the protocols to ensure the safety of participants and interviewers was adequately protected.

## Results

We collected data from 2,000 households in 100 clusters, distributed across Iraq's 18 governorates. After removing the two clusters previously mentioned, the total household count was 1,960, with an average of 5.34 members per household. The study population was distributed similarly to Iraq's estimated total 2009 population as reported by COSIT, which based its estimate on projections from the 1997 census for the 15 southern governorates and on the 1987 census for the three Kurdish governorates. We compared the proportion of our sample to the proportion of the total population in each governorate as reported by COSIT, and derived an index of dissimilarity of 14% [50]. The percent of recorded deaths with missing “cause of death” data is small. See Table 1.

**Table 1:** Sample size and counts of household members and siblings in the University Collaborative Iraq Mortality Study, by governorate.

Governorate	Estimated 2009 COSIT Population	Proportion of Total Iraq Population	Number of Clusters	Proportion of Sample	Number of Adults Reporting on Siblings	Number of Unique Siblings Reported	Number of Household Heads Reporting on Household Members	Household Members at Time of Survey	Percent of Siblings Missing Cause of Death^a^
Al-Anbar	1,483,359	0.045	7	0.07	375	2,540	140	990	0.12%
Al-Basrah	2,405,434	0.080	8	0.08	335	1,908	160	884	0.05%
Al-Muthanna	683,126	0.022	1	0.01	61	371	20	142	0.27%
Al-Najaf	1,221,228	0.037	2	0.02	68	398	40	200	0.51%
Al-Qadisiya	1,077,614	0.035	4	0.04	234	1,366	80	580	0.15%
Al-Sulaimaniya	1,784,853	0.048	7	0.07	291	1,822	139	663	0.22%
Babylon	1,729,666	0.054	3	0.03	154	877	61	353	0.23%
Baghdad	6,702,538	0.224	23	0.23	1,066	5,442	460	2,347	0.17%
Diala	1,371,035	0.043	5	0.05	183	1,050	100	463	0.19%
Duhouk	1,072,324	0.030	2	0.02	98	606	40	284	0.00%
Erbil	1,532,081	0.046	9	0.09	357	2,055	180	803	0.10%
Kerbela	1,013,254	0.031	2	0.02	109	607	40	221	0.33%
Kirkuk	1,325,853	0.040	2	0.02	98	502	40	201	0.00%
Maysan	922,890	0.031	3	0.03	118	704	60	311	0.00%
Ninevah	3,106,948	0.101	13	0.13	467	2,846	260	1,298	0.04%
Salah Al-Deen	1,337,786	0.039	3	0.03	111	669	60	312	0.30%
Thi Qar	1,744,398	0.058	3	0.03	105	672	60	308	0.00%
Wasit	1,150,079	0.036	3	0.03	120	730	60	310	0.00%
All governorates	32,104,988	100%	100	100%	4,350	25,165	2,000	10,670	0.13%

Population data from COSIT.

a As a check of validity.

Interviewers reported that 24 households refused to participate in the study, and five households were not interviewed because of hostile or threatening behavior (resulting in a 98.55% response rate). This low refusal rate is not uncommon for surveys in similar countries [51]. An additional 188 buildings were occupied by a business or other establishment, rather than a household, and four previously selected start dwellings were found to have been destroyed. In all these cases, replacement households were chosen using our established study protocol, to ensure total households numbered 20 per cluster.

### Household Survey Results

The majority of the heads of the 1,960 households were male (85%). At the beginning of the first time period (January 2001), 1,313 of these households were already established, and contained approximately 6,455 members. A total of 2,735 births and 383 deaths were reported during the study period. Sex and cause of death were reported for 98.4% of deaths (*n* = 377). Of 10,467 household members at the survey date, 50% were male, and 42% were children under the age of 18 y. On average, households had existed as a unit for a mean of 19.9 y (and a median of 17 y) at the time of interview. The crude birth rate was 35.5 per 1,000 persons in 2001, and 32.7 in 2010. Estimated wartime crude death rates ranged from 2.0 per 1,000 person-years (PY) for females in 2011 to 7.9 for males in 2005–2006; pre-war crude death rates (2001–February 2003) were 2.1 per 1,000 PY for females and 3.7 for males.

Respondents attributed 19% of household deaths to war-related violence (*n* = 72) and named a responsible entity for 79% (*n* = 59) of those deaths. See Table 2 for violent deaths reported by type and responsible party.

**Table 2:** Counts of reported violent deaths by responsible party and by cause, by year and source of report, as collected in the University Collaborative Iraq Mortality Study.

Category	Sub-Category	Time Period			Percentage for 2003–2011
		2003–2006	2007–2011	2003–2011	
**Responsible party for violent deaths**					
	**Source: household reports**				
	All responsible parties	48	27	75	100%
	Coalition forces	22	4	26	35%
	Militia	13	11	24	32%
	Criminals	5	3	8	11%
	Iraq forces	0	1	1	1%
	Other/unknown	8	8	16	21%
	**Source: sibling reports**				
	All responsible parties	121	61	182	100%
	Coalition forces	42	7	49	27%
	Militia	52	29	81	44%
	Criminals	9	5	14	8%
	Iraq forces	0	5	5	3%
	Other/unknown	18	15	33	19%
**Cause of violent deaths**					
	**Source: household reports**				
	Gunshot	30	17	47	63%
	Car bomb	5	4	9	12%
	Airstrike	5	0	5	7%
	Other explosion	3	4	7	9%
	Other war injury/don't know	5	2	7	9%
	**Source: sibling reports**				
	Gunshot	63	35	98	54%
	Car bomb	8	9	17	9%
	Airstrike	21	3	24	13%
	Other explosion	13	7	20	11%
	Other war injury/don't know	16	7	23	13%

Survey of 1,960 households in Iraq between May and July of 2011, with death due to war-related injury recorded for 75 deaths among household members, and 182 deaths among adult siblings for the period March 1, 2003–June 30, 2011. (Percentages may not sum to 100% due to rounding.)

Despite receiving the most press coverage, explosive devices were not the leading proximate cause of death among war casualties—rather, gunshots were [52],[53]. Gunshots were reported to cause 63% of violent deaths; car bombs, 12%; and other explosions, 9%. Gunshot deaths were most common for the period March 1, 2003–December 31, 2008, and dropped precipitously thereafter.

US-led coalition forces were reported to be responsible for the largest proportion of war-related violent deaths (35%), followed by militia (32%). While militia were reportedly responsible for the most adult male deaths in the sibling survey, coalition forces were reportedly responsible for killing the most women.

**Figure 1:**
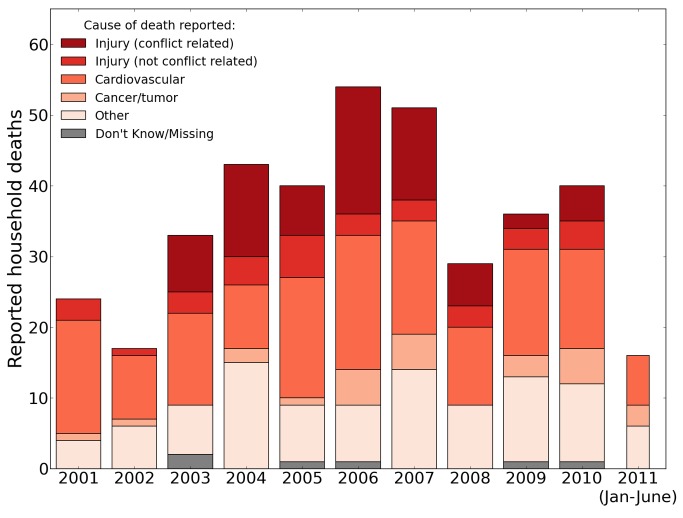
Raw number of household deaths by year and cause, 2001–2011, reported by the University Collaborative Iraq Mortality Study. Counts of deaths reported by respondents to the household mortality questionnaire, by year and cause. The survey concluded July 2, 2011, so the final bar reflects data for only half of the year.

Cardiovascular conditions were the main cause of nonviolent death, accounting for 47% of nonviolent deaths over the entire study period (*n* = 146). Other common sources of nonviolent deaths included chronic illnesses (11%, *n* = 35), infant or childhood deaths other than injuries (12.4%, *n* = 38), non-war injuries (11%, *n* = 33), and cancer (8%, *n* = 26). See Figure 1 for the number of household deaths by year and cause, 2001–2011.

At the end of the interview, surveyors asked to see death certificates for reported deaths, and were shown certificates for 283 (74%) of the deaths. The certificate was reported to be present but not seen by the surveyor in an additional 17% of deaths. Death certificates were queried to confirm deaths rather than to establish the cause of death. Iraqi team members believed family cause-of-death reports were likely to be more accurate than cause of death on certificates, as the true cause of death was often not given during times of intense insecurity. The percentage of violent deaths reported by households with death certificates available did not differ substantially from those without. The percentage of households reporting deaths that had death certificates, either shown or claimed (91%), was identical in the 2006 and 2011 studies, indicating that the availability of death certificates remained high throughout the war.

**Figure 2:**
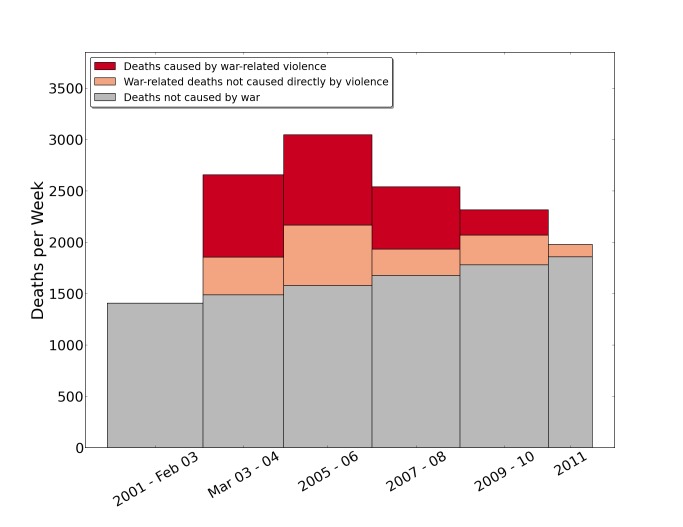
Estimates of numbers of deaths per week in Iraq for 2-y intervals, 2001–2011, by cause as reported by households in the University Collaborative Iraq Mortality Study. National estimate of deaths in Iraq between 2001 and 2011. Crude death rates were estimated separately within 2-y blocks (the first two time intervals are not strictly 2 y long, in order to align the first interval dividing point with the start of the war in March 2003; the survey concluded July 2, 2011, so the final bar reflects only half of the year). The counterfactual (had there been no war) estimate shows the predicted death counts if crude death rates had remained at their average level from 2001–2002 during the war and occupation (in gray). War-related, but not violent, deaths above the normal baseline are in the salmon-colored area. War-related violent deaths are portrayed in red.

The wartime crude death rate in Iraq was 4.55 per 1,000 PY (95% UI 3.74–5.27), more than 0.5 times higher than the 2.89 ((95% UI 1.56–4.04) death rate during the 26-mo period preceding the war. By multiplying those rates by the annual Iraq population [43], we estimate total excess Iraqi deaths attributable to the war through mid-2011 as about 405,000 (95% UI 48,000–751,000). We illustrate both expected and excess deaths per week in Figure 2.

**Figure 3:**
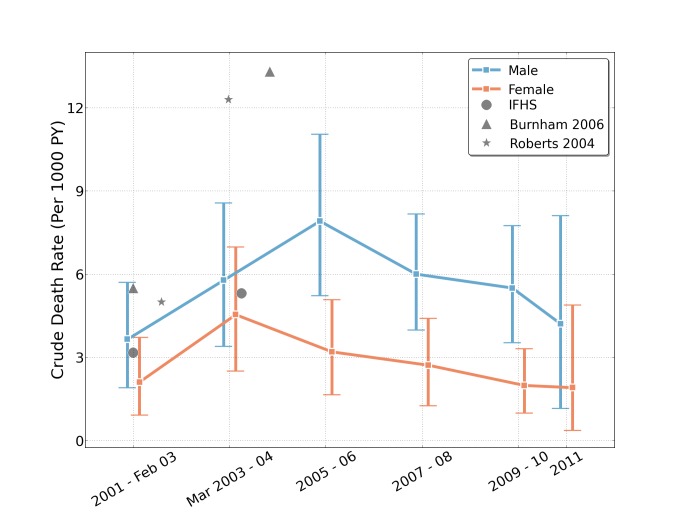
Estimated crude death rates (and 95% uncertainty intervals) by sex and 2-y intervals from household reports in the University Collaborative Iraq Mortality Study, with plotted estimates from other studies. Estimates of the differences in male and female death rates in households over the period 2001–2011, based on interviewing 1,960 household heads across Iraq. Mortality estimates from other studies are plotted as point estimates for comparison purposes [30]–[32]. The first two time intervals are not strictly 2 y long, in order to align the first interval dividing point with the start of the war in March 2003; the survey concluded July 2, 2011, so the final bar reflects only half of the year. PY, person-years.

In the post-invasion time period, the sex ratio of violent deaths was 8.5 males to every one female, compared to 2.1 males to each female for deaths of all causes during the same time period. See Figure 3, which also plots estimates from other studies.

### Sibling Survey Results

We collected data from 4,287 adults in the 1,960 households who reported on the vital status (alive or dead) of their 24,759 siblings. The respondents reported having an average of 5.8 siblings, 0.6 of whom had died (2,531 dead). Of total sibling deaths, 65% (*n* = 1,641) were male. The sibling history questionnaire provided cause of death for 94% of sibling deaths. The highest proportion of all sibling deaths occurred in Baghdad (24.7%, *n* = 625), slightly higher than the 22% of the population that Baghdad comprised in our study, and the lowest in Al-Najaf (1.15%, *n* = 29), somewhat below its 2% of the population in the study. Only 2.2% of sibling deaths were reported to occur outside Iraq (*n* = 51), although in cases where an entire sibship migrated, there would be no remaining siblings in Iraq to report on deaths.

The ICSS method generates age-, time-, and sex-specific estimates of adult mortality rates. In applying the zero-survivor correction, 3.7 missing siblings were added to the dataset.

After applying the death rates found in our sample of Iraqi adults to the age-, time-, sex-specific UN population estimates, we estimate the total number of deaths among adults aged 15–60 y, from March 1, 2003, to June 30, 2011, to be approximately 376,000 (95% UI 308,000–441,000). Of those, 187,000 (95% UI 64,000–288,000) were excess deaths (caused directly or indirectly by conflict), with 132,000 (95% UI 89,000–174,000) war-related violent deaths.

**Figure 4:**
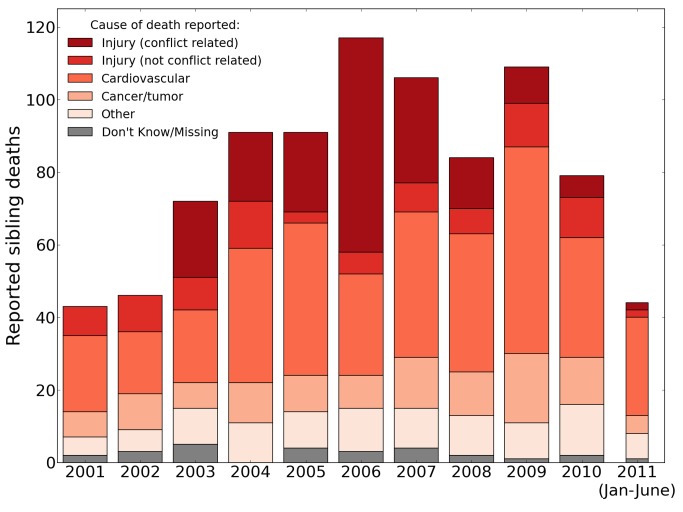
Raw number of adult deaths by year and cause, 2001–2011, reported by the University Collaborative Iraq Mortality Study. Counts of deaths reported by respondents to the sibling survival questionnaire, by year and cause. The estimates use the ICSS method to correct for survival bias [33]. The survey concluded July 2, 2011, so the final bar reflects data for only half of the year.

Figure 4 portrays the raw number of adult deaths (reported by siblings) by year and cause, 2001–2011, illustrating findings similar to the pattern of household deaths. There were 295 sister deaths between 2003 and 2011, of which 277 (93.9%) were reported to be from a cause other than war-related-violence. These deaths were primarily attributed to cardiovascular causes (*n* = 142, 48%) and cancer (*n* = 52, 18%). Among brother deaths for the same period, 67% were reported to be from a cause of death other than war-related violence. As with sisters, the primary reported causes of nonviolent deaths were cardiovascular disease (36%, *n* = 180) and cancer (10%, *n* = 48).

**Figure 5:**
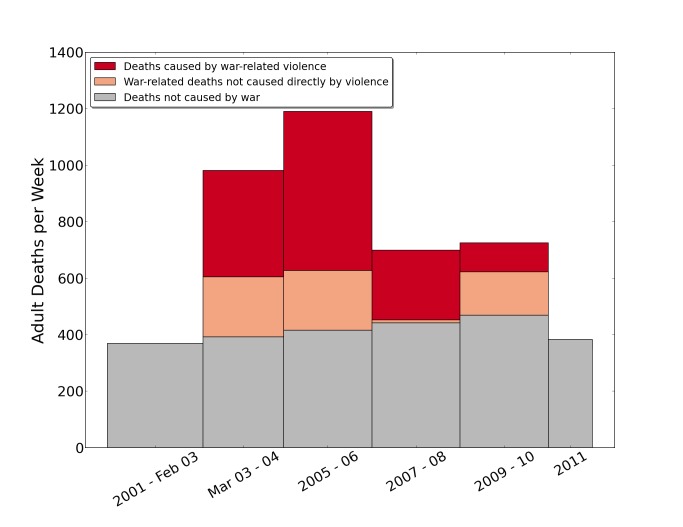
Estimates of numbers of adult deaths per week in Iraq for 2-y intervals, 2001–2011, by cause as reported by siblings in the University Collaborative Iraq Mortality Study. National estimate of deaths in Iraq between 2001 and 2011. Age-specific mortality rates were estimated separately within 2-y blocks (the first two time intervals are not strictly 2 y long, in order to align the first interval dividing point with the start of the war in March 2003; the survey concluded July 2, 2011, so the final bar reflects only half of the year). The counterfactual (had there been no war) estimate shows the predicted death counts if crude death rates had remained at their average level from 2001–2002 during the war and occupation (in gray). War-related, but not violent, deaths above the normal baseline are in the salmon-colored area. War-related violent deaths are portrayed in red. The estimates use the ICSS method to correct for survival bias [33].

Figure 5 provides estimates of numbers of adult deaths per week in Iraq for 2-y intervals (2001–2011) by cause, illustrating the rising expected number of deaths per week over the decade as the population increased, and with excess war-related deaths attributable to both violent and nonviolent causes. For example, in the period 2005–2006, there were about 766 excess deaths per week, with the majority of these (72.6%) attributable to war-related violence.

**Figure 6:**
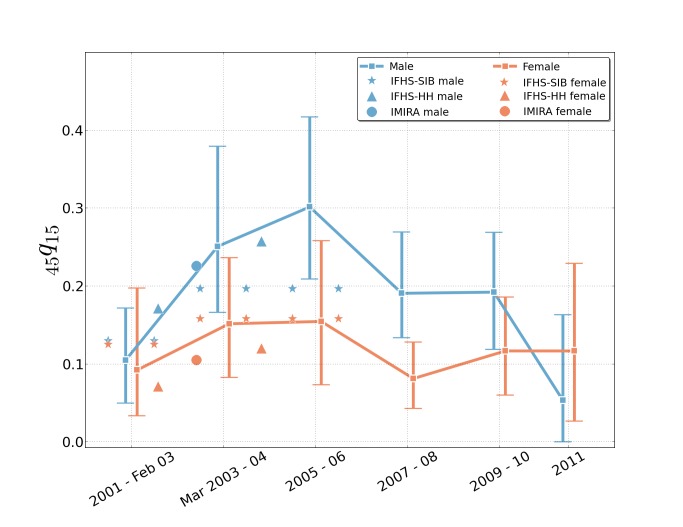
Estimates of the probability of dying between age 15 and age 60-y intervals, 2001–2011, from sibling histories as reported in the University Collaborative Iraq Mortality Study, with plotted estimates from other studies. Estimates of adult mortality risk (_45_ *q*_15_, the risk of dying between the ages of 15 and 59 y), over the period 2001–2011, based on the sibling history survey. The first two time intervals are not strictly 2 y long, in order to align the first interval dividing point with the start of the war in March 2003; the survey concluded July 2, 2011, so the final bar reflects only half of the year. The estimates use the ICSS method to correct for survival bias [33]. Mortality estimates from other studies are plotted as point estimates: IMIRA (part of the Iraq Living Conditions Survey) [28], the IFHS household survey (IFHS-HH), and the IFHS sibling survey (IFHS-SIB) [32].

Figure 6 portrays the probability of dying between age 15 and age 60 y in 2-y intervals (2001 to 2011), estimated from sibling histories—along with comparisons to adult probability of death generated by other population-based sampling studies. Prior to the invasion, the probability of dying before age 60 y among females who had achieved 15 y of age was 0.093 (95% UI 0.034–0.197), and among males it was 0.105 (95% UI 0.050–0.172). At the peak of the war, in 2006, this probability for females was 0.155 (95% UI 0.074–0.258), which declined to 0.117 (95% UI 0.027–0.229) by 2011. For adult males, the risk of dying before age 60 y was 0.302 (95% UI 0.209–0.417) at the peak of the war (2005–2006), and it declined to 0.054 (95% UI 0.000–0.163) by 2011. Among adults, then, the risk of death rose 1.7 times higher for women and 2.9 times higher for men between the pre-war period and the peak of the war in 2006. The peak of conflict-related deaths was in 2006, corresponding to the peak of flight from Iraq, and the peak of internal displacement [54].

### Baghdad Sample Selection Overlaid on Conflict Data

**Figure 7:**
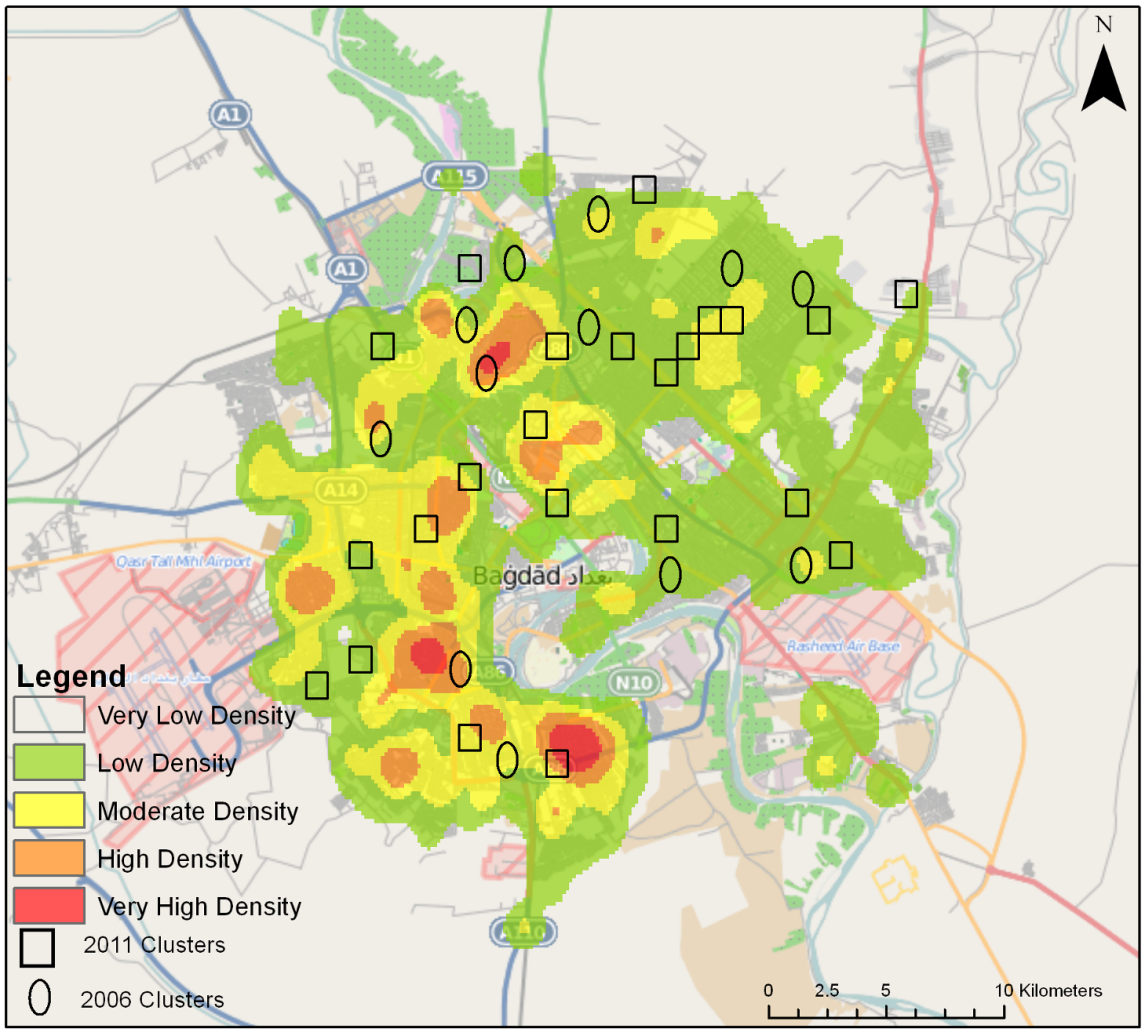
Density of civilian deaths in Baghdad, with the cluster locations of the University Collaborative Iraq Mortality Study as well as 2006 cluster locations of a previous study [30]. The density map was generated using a kernel density estimation of civilian deaths from the Wikileaks Iraq War Logs release [55]. The kernel density estimation provides a smoothed surface from a point pattern that represents spatial variation in the density of events, in this case civilian deaths. This allows for a crude visual analysis of the variation of events across space in Baghdad as well as the relation of 2006 and 2011 cluster locations to the density of civilian deaths. Analysis is based on geolocated data of all civilian deaths by any means reported in the Wikileaks Iraq War Logs data from 2004 to 2009.

For illustrative purposes, we generated a density map portraying civilian deaths. We overlaid Baghdad survey cluster locations in relation to the spatial variation of civilian deaths reported elsewhere for the period 2004–2009 (Figure 7) [55]. The figure also gives the locations of the clusters from a 2006 survey [30] and our 2011 survey. The map shows no specific pattern of cluster selection in relation to reported civilian deaths.

### Migration Effects

United Nations sources estimate that about 1.7 million Iraqis have migrated abroad since the start of the war [56], while other estimates put the number at closer to 2 million [57] or even 2.4 million [58]. In addition, more than 1.3 million persons have been displaced within Iraq, most fleeing after 2006 [59],[60]. In a 2010 national population survey, one in six households had reportedly moved in the previous 5 y, with “escaping violence” the most common reason [61]. Refugee and internally displaced persons estimates are probably low, as a portion of refugees do not register with the United Nations High Commissioner for Refugees or the International Organization for Migration [46]. Given an average household size of 5.34—as found in the current study (compared with 6.9 in a previous 2006 study [30])—and an emigrant population of 2 million, this would yield 374,532 external refugee households. One study in Syria estimated that 14.9% of Iraqi refugee households had experienced at least one death [46]. Conservatively, assuming that only 15% of the emigrant households experienced a death, our migration adjustment would add more than 55,000 deaths to the total generated by our household survey (calculation: 2 million migrants/5.34 per household = 374,532 households; those households×0.149 experiencing deaths = 55,805 total deaths attributable to external migrants).

### Total Estimate

Our household survey produced death rates that, when multiplied by the population count for each year, produced an estimate of 405,000 total deaths. Our migration adjustment would add an additional 55,805 deaths to that total. Our total excess death estimate for the wartime period, then, is 461,000, just under half a million people.

## Discussion

We estimate about half a million excess deaths occurred in Iraq following the US-led invasion and occupation (March 2003–2011). This estimate is derived from reports of deaths by respondents in our nationally representative survey of 2,000 households in 100 clusters, and adds a correction for deaths that would have been reported by households that emigrated. Our household data indicate that the wartime crude death rate in Iraq was 4.6 per 1,000 PY, more than 0.5 times higher than the death rate during the 26-mo period preceding the war. We were also able to estimate the risk of death among adults, both before and during the war, by asking all adults in the household about the deaths of their siblings, and learned that the risk of death rose 0.7 times higher for women and 2.9 times higher for men between the pre-war period and the peak of the war. There were an estimated 766 excess (war-related) adult deaths per week when the war was taking its highest casualties, with about 70% of these attributable to war-related violence.

Five previous studies of mortality in Iraq were conducted over the course of the war using household surveys. Roberts et al. [31] estimated 12.3 deaths per 1,000 PY (for the period 2003–2004). That finding was reasonably similar to the rate obtained by the study by Burnham et al. [30] conducted 2 y later (2003–2006), which reported 13.2 deaths per 1,000 PY. The IFHS [32] conducted during a similar period (2003–2006) reported a lower crude death rate (5.31 per 1,000 PY), although this survey failed to collect primary data from 115 high-violence clusters (of 1,086), and instead imputed missing data for these clusters from Iraq Body Count (http://www.iraqbodycount.org/) figures. The Iraq Living Conditions Survey [28] conducted in the spring of 2004 attempted to count war-related deaths for the period March 20, 2003–May 30, 2004 (estimated at between 18,000 and 29,000) and war-related chronic illnesses (200,000), but did not report an all-cause death rate. At the high end of estimates, an Opinion Research Business poll [29] in 2007 estimated a violent (not all-cause) mortality rate of 10.3 per 1,000 PY for all but three governorates (Kerbela, Al-Anbar, and Erbil). As an alternative to conducting household surveys, the Iraq Body Count used media and other accounts to simply tally civilian war fatalities, arriving at a total of about 116,475 (or about 0.4 deaths per 1,000 PY as of February 2013). We provide comparisons of some of these rates in Figures 3 and 6.

We have three hypotheses for the low crude death rates we found in comparison to three previous retrospective mortality surveys: sampling differences, recall bias, and other non-sampling errors, and/or reporting problems related to migration. Our study used different sampling methods than Roberts et al. [31], Burnham et al. [30], and IFHS. Not only did we sample at least twice the number of clusters as Roberts et al. and Burnham et al.—albeit with the same sample size—we also selected the sample using a more sophisticated randomization approach. Our methods may have avoided biases that served to overrepresent deaths in the other two studies. In contrast to IFHS, we skipped only one cluster for security reasons, and did not substitute Iraq Body Count data, which we know underrepresent death rates. The long recall period required of participants in this study likely contributed to underreporting of deaths, and in the setting of a country with increasing sectarian divisions, some people may have been unwilling to report deaths, as well. The war has also caused wide-scale redistribution of Iraq's population, both internally and externally; we know we missed the families that migrated out of the country, and likely missed a representative proportion of internally displaced people as well. We know the earlier census data did not capture these movements, and our sample was selected using those data. It is highly likely that households experiencing more violence were more likely to migrate, thus serving to reduce our death rates using the retrospective mortality survey method.

The gold standard for measuring conflict-related mortality is prospective active surveillance, with real-time data collection of mortality events as they occur [62],[63]. International initiatives to commence these methods prior to the outbreak of war have been recommended [64], and could be initiated now for the several anticipated or emerging armed conflicts. Failing that, retrospective surveys are the next best approach, despite their shortcomings (which include delays in analysis and reporting, large confidence intervals, lack of good baseline data for comparison purposes, and the inability to capture varying results by sub-region using feasible sample sizes). Body counts based on passive surveillance are the least reliable of methods [62].

Our study has limitations. National systems of census count and vital registration are typically disrupted during war, making it difficult to use recent measures for a denominator population. We relied on Iraq's decades-old census reports, as more recently projected by the national population authority (COSIT). These are obviously imperfect. Our sub-analysis of Baghdad clusters (Figure 7) and the comparison of our sample to the COSIT census distribution by governorate (with a 14% index of dissimilarity) indicates our sample was representative. Any cluster sample, however, is likely to miss sequestered enclaves of various types, some of which may have had particularly low or high mortality. Our sample likely did not include a good representation of the 1.1 million people who were living in camps or buildings as internally displaced people [60]. Additionally, we replaced one start household that no longer existed, and when a household was vacant after two visits we replaced it; these could have led to sampling bias. Verbal autopsies were not done, and thus family reports on causes of death were not validated.

Retrospective reports of death by household members and siblings require survivors to make accurate accounts. Threats to validity of estimates from household and sibling reports include the lack of survivors (due to household dissolution or migration or no surviving siblings), recall bias, unwillingness to report, and sampling error.

To address survivor bias, we attempted to estimate deaths that would have been reported by household members that had migrated. The sibling method we used also includes a correction for survival bias. We assessed the role of recall bias at length (see Text S2 for details), and concluded that our results were robust to this potential problem, provided such bias affected both pre-war and post-war reporting periods.

Most excess deaths (above pre-war rates) were attributable directly to violence, primarily from gunshots, car bombs, and explosions. Cardiovascular conditions were the principal cause of about half of nonviolent deaths. War-induced excess deaths not caused by violence would include those caused by diversion of the health system to a focus on crisis care, interruption of distribution networks for crucial supplies, and the collapse of infrastructure that protects clean water, nutrition, transportation, waste management, and energy. Further, war contributes to a climate of fear, humiliation, and interruption of livelihoods that undermines health [65]–[68].

The pattern of mortality we observed with both our household and sibling methods correlates with media accounts of how the violence rose and ebbed over the years of the war. Deaths increased to twice expected levels at the onset of the war, plateaued briefly at the end of 2003, then rose again to a new peak in 2006. Thereafter, deaths dropped until 2008, when they leveled off, and then rose again slightly just before the time of our data collection in 2011.

The number of events recorded by the households we visited was relatively small, yet it generated rates that appear large when magnified to the national population; that is the nature of this method. On the other hand, we did not adjust for world secular trends of declining mortality, therefore probably understating the number of excess deaths over this long conflict.

Although the US military initially denied tracking civilian deaths, 2011 Wikileaks documents revealed that coalition forces did track some noncombatant deaths. The emergence of the Wikileaks “Iraq War Logs” reports in October 2010 [69] prompted the Iraq Body Count team to add to its count, but a recent comparison of recorded incidents between the two databases revealed that the Iraq Body Count captured fewer than one in four of the Iraq War Logs deaths [70]. One important reason for the discrepancy is that small incidents are often missed in press reports. For example, when asked why the assassination of a medical school dean in Baghdad did not merit reporting, Tim Arango (of the *New York Times*) stated in personal correspondence to AH in April 2011, “Unfortunately there are numerous assassinations every day, and we cannot cover them all.”

The deaths of citizens swept up in the conflict are seldom commemorated [71], and yet memorializing and reconciling these casualties has been found to be important for creating a peaceful post-conflict society [72]–[74]. Those who attempted to predict the mortality consequences of an impending invasion of Iraq in 2002 under-projected the death count [75], because methods for this type of assessment remain too crude. Estimates of mortality in the final stages of this protracted war suffer from methodological problems as well, because of complex population shifts [54]. Our contribution has been to use one nationally representative sample to collect both household and adult sibling mortality data. Researchers should continue to refine methods to count the mortality effects of conflicts.

The American Public Health Association has adopted a policy encouraging governments to conduct health impact assessments prior to making policy decisions, such as entering into war [76]. When researchers can refine methods to project death counts in advance, as well as to measure total deaths incurred as wars conclude, the public can make wiser decisions about the costs of entering into armed conflict. An authoritative worldwide body could assemble scholars to perfect these methods.

## Supporting Information

Figure S1 **Figure illustrating sample selection.**
(DOCX)

Manual S1 **Field manual for data collection personnel.**
(DOC)

Questionnaire S1 **Questionnaire used by data collection personnel.**
(DOCX)

Text S1 **Additional methods.**
(DOCX)

## Abbreviations

COSIT: Iraqi Central Statistical Organization
ICSS: improved corrected sibling survival
IFHS: Iraq Family Health Survey; IMIRA, Iraq Multiple Indicator Rapid Assessment
PY: person-years
UI: uncertainty interval
