# Supplementary material for: Mortality in Iraq Associated with the 2003–2011 War and Occupation: Findings from a National Cluster Sample Survey by the University Collaborative Iraq Mortality Study
Source: PLoS Med. 2013 Oct 15;10(10):e1001533. doi: 10.1371/journal.pmed.1001533 (PMC3797136; doi:10.1371/journal.pmed.1001533)
Supplement: Figure S1 — Figure illustrating sample selection. (DOCX) [file pmed.1001533.s001.docx]

### Supplemental Figure 1: Density: Map of Iraq illustrating clusters (first sampling stage)


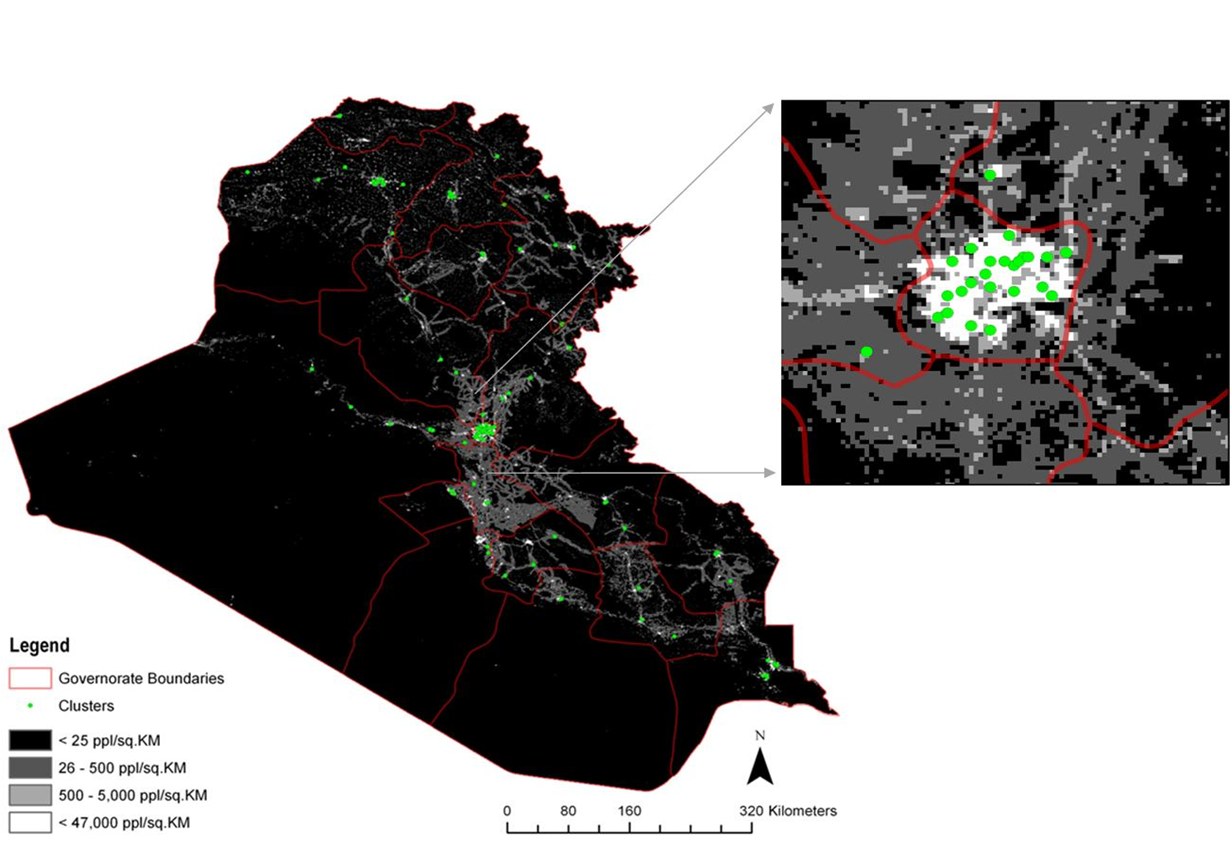


*Illustration of the first stage of a two-stage cluster sampling method using gridded population data, a GIS, and Google Earth^TM^ imagery in a population-based mortality survey in Iraq, as described elsewhere.*

Citation: Galway LP, Bell N, Al- Shatari SAE, Flaxman A, Rajaratnam J, Weiss WM, et al. A two-stage cluster sampling method using gridded population data, a GIS, and Google EarthTM imagery in a population-based mortality survey in Iraq. Int J of Health Geographics. 2012.
