# Supplementary material for: Mortality in Iraq Associated with the 2003–2011 War and Occupation: Findings from a National Cluster Sample Survey by the University Collaborative Iraq Mortality Study
Source: PLoS Med. 2013 Oct 15;10(10):e1001533. doi: 10.1371/journal.pmed.1001533 (PMC3797136; doi:10.1371/journal.pmed.1001533)
Supplement: Manual S1 — Field manual for data collection personnel. (DOC) [file pmed.1001533.s002.doc]

**Field Manual**

[
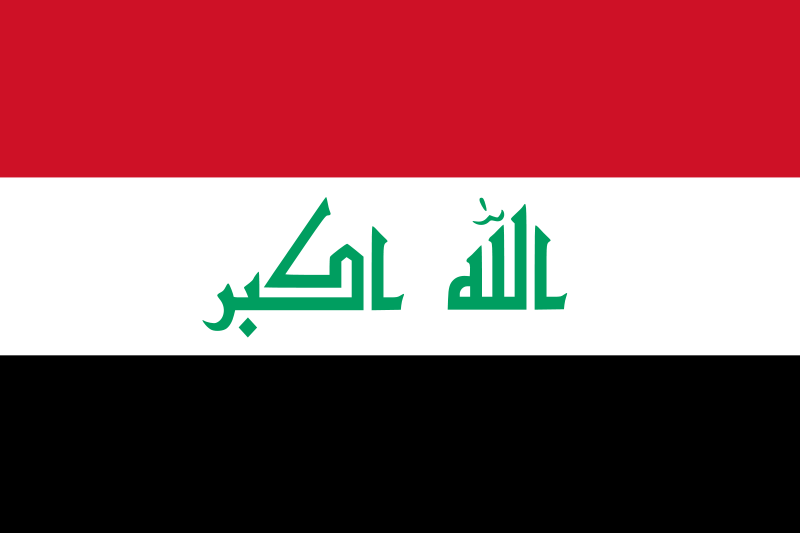
](http://upload.wikimedia.org/wikipedia/commons/f/f6/Flag_of_Iraq.svg)**2011 Iraq Population Demographic Survey**

**18 April 2011**

**Al Mustansiriya University**

**University of Washington**

**Johns Hopkins University**

**Simon Fraser University**

**2011 Iraq population demographic survey**

**field manual**

## Version of 18 April 2011

Version of 18 April 2011 [2](#__RefHeading___Toc199977955)

Overview [3](#__RefHeading___Toc199977956)

Goal of the study [3](#__RefHeading___Toc199977957)

Reasons for the Study [3](#__RefHeading___Toc199977958)

Data collection [4](#__RefHeading___Toc199977959)

The Questionnaire [4](#__RefHeading___Toc199977960)

What is a household? [4](#__RefHeading___Toc199977961)

2. Births and Deaths and migration [5](#__RefHeading___Toc199977962)

3. Sibling Questionnaire [5](#__RefHeading___Toc199977963)

Choosing neighborhoods and households [5](#__RefHeading___Toc199977964)

How to approach a household [7](#__RefHeading___Toc199977965)

Who to interview? [8](#__RefHeading___Toc199977966)

Questionnaire script [9](#__RefHeading___Toc199977967)

Understanding the questionnaires (question by question review) [9](#__RefHeading___Toc199977968)

Questionnaire [9](#__RefHeading___Toc199977969)

Entry of data [14](#__RefHeading___Toc199977970)

Qualitative data [14](#__RefHeading___Toc199977971)

The study team [15](#__RefHeading___Toc199977972)

Roles and responsibilities within the field team [15](#__RefHeading___Toc199977973)

Project Manager: [15](#__RefHeading___Toc199977974)

Interviewer [17](#__RefHeading___Toc199977975)

Quality Checks [18](#__RefHeading___Toc199977976)

Communications [18](#__RefHeading___Toc199977977)

Record Keeping [19](#__RefHeading___Toc199977978)

The consent form [19](#__RefHeading___Toc199977979)

Maintaining confidentiality [19](#__RefHeading___Toc199977980)

Security [19](#__RefHeading___Toc199977981)

Safety rules: [20](#__RefHeading___Toc199977982)

Confidentiality [20](#__RefHeading___Toc199977983)

Documents [20](#__RefHeading___Toc199977984)

Phone numbers [20](#__RefHeading___Toc199977985)

Trust [20](#__RefHeading___Toc199977986)

Tracking [20](#__RefHeading___Toc199977987)

Driver and movement [20](#__RefHeading___Toc199977988)

Household recruitment script (see Arabic translation) [21](#__RefHeading___Toc199977989)

# Overview

## Goal of the study

The goal of this study is to examine the demographic pattern changes across Iraq before and after March of 2003, specifically the patterns of births, deaths, and migration

## Reasons for the Study

During times of peace and order in reasonably prosperous countries, demographic changes in a population are estimated from civil registries, national census, hospital records, and university-based research. In times of armed conflict, these systems tend to collapse. For this reason, changes have to be estimated using sampling methods. When retrospective methods must be used, surveyors face threats to validity, including population movements, birth rate changes, and lost reporting chains. Documenting what happens to populations in war is a way to bring awareness of the vulnerabilities that people experience during conflict and promote ways to improve protection.

Several previous surveys have made estimates of violent and non violent deaths in Iraq. Since those surveys were done, new methods have been developed and tested. The most important new measure is called the Sibling Survival method. This collects information on the brothers and sisters of all adults in each household visited. The study will also use the traditional measures of household births and deaths. Combining these traditional and new methods, we propose to estimate deaths in Iraq before and after March 2003.

There is a paper attached to this manual that illustrates an example of Sibling Survival research.

We are fortunate to have an experienced field team, as well as people experienced in both the traditional and the new measurement methods.

***Methods of this study***

This will be a survey from randomly selected households in 100 sites (clusters) across Iraq. For this survey we define a household as a group of people who regularly eat and sleep together in a building with a separate entrance and who share a kitchen. They need not be related, so this makes them different from the definition of a “family.” We will collect information about 1) household residents and 2) siblings of all adults in each household. One advantage of the Sibling method is that we can get relatively accurate information about many people besides those living in the household.

***Accuracy of the information recorded***

For the correct conclusions to be made from this study, the accuracy and completeness of the data are the most important parts of the study. If data are recorded incorrectly, or if some people in the household are missed, then the results will not be accurate. Getting information from only those easily found will result in a bias, and the results of the survey will not reflect the actual situation. This survey must be as complete and accurate as possible with information on ALL persons living in the household being collected. This is especially important for the siblings of ALL adults present in the household.

Everyone, including the interview team and the households, will have different views about Iraq’s war deaths and injuries. This is a scientific study, so please set aside you personal views and just seek the information without letting any personal views intervene. Personal views can produce bias, which we must avoid at all costs!!

To emphasize the importance of carefulness and completeness, all members of the research team are signing a statement saying they will complete their part of the study with as much care and accuracy as possible, and they recognize this as their responsibility to this study.

# Data collection

## The Questionnaire

This study will collect household and adult sibling information. The first step is completing the household listing.

1. *Household listing*

### What is a household?

We’ll ask household heads to answer the household survey, where possible, but if not, the senior adult in the household can answer. This senior person could be the most important or oldest or most knowledgeable person in the household after the head of household.

The household listing records the sex and age of all residents of the household. A household is comprised of people who have been living in the household and who meet the study definition of a member of the household. List adults first, starting with the head of household or the senior adult, whom ever is the person you are interviewing for household information. Please note whether they need to be interviewed for the sibling survey. List children second by age at completed birthday. For children under age one, use months.

***Note:*** The **head of household or senior household member** should be interviewed for the *household survey* of birth and death listings. **Every** person aged 18 and older (15 and older for any woman who has been married), normally resident in the household should be interviewed for the *sibling history*. Individuals should be interviewed separately--not as a group, and in private spaces wherever possible.

If someone wonders whether to list a resident as a present member of their household, we want you to only include only those who have been there since New Years of 2011 or who have recently joined the household with the intent of residing there for a period of four months or more. For example, children recently born or brought into the household and/or men or women recently married into the household would be considered members of the household.

You will ask when a household was formed. This could be when the oldest members of the household were married, thus starting this household, or it could be when the person being interviewed moved into the household. A household includes everyone who was there at the moment of household formation (say, a couple get married and move to a new location), or January 1, 2001, whichever comes later. For example, if a couple was married in 2002, we would only be interested in the household as it consisted as of from 2002 onward. However, if a household were formed in 2005 by marriage, that is the “start date” for this household, and we would not collect information before that time. If a son got married and moved into the household of his mother, then the household to consider is that of his mother which would probably go back before 2001, though we would be interested only in information about births and deaths from January 2001 onward.

If the next “household” you face is an apartment building or a place where multiple family units reside, you should ask the supervisor to help you, but you would collect information from all households within that apartment building. The same defineton of a household would apply.

### 2. Births and Deaths and migration

The information you record concerns births and deaths since 2001, and the number of moves to a new city. Only record a death if the person was living in the household regularly at the time of death, and had been doing so for at least six months before the death. This means someone who occasionally visited the household and died would not be included. But information would be collected for someone who generally ate and slept in the household before their death. An unmarried child away at school or in the military, but who comes home for holidays would be a member of the household, but a son or daughter who was staying mostly in the household of another relative while working or attending school would not be considered a member of the household. Someone who was in prison could be considered a member of the household, if they did not have some other home that they would go to on discharge from prison.

This section also asks about how many places the key informant has lived since January 2001

### 3. Sibling Questionnaire

This sibling questionnaire gathers information about all the brothers and sisters (siblings) of **each adult** in the household. A sibling is someone who shares the same biological (natural born) mother. If there are two or more siblings in the household you should select one. To choose which sibling to survey when they both (or several) live in the same house or cluster, select whichever one is actually present, or if all are present, choose the sibling with the nearest next birthday to this date of the visit. On the cover sheet you should note which were siblings and not interviewed.

If there is another sibling in another household in the same cluster of households, this person may be interviewed, but please note this on the cluster cover sheet so this fact may be considered at time of the analysis.

Do not interview someone who is just visiting the household. If it is unclear whether this person is visiting, include the person if they have been in this household since 1 January of this year or moved here with the intent to stay. It is very important that **all adults**, that is all persons at least age 18, be interviewed. If they are not present at the time of the interview, please find out what time is best for you to return, or telephone them on a cell phone and obtain the information for the questionnaire over the mobile phone. Often two or three “call back visits” may be required to collect information from all the adults in the household.

The mother of adult siblings in the home can report on her own children (and, obviously, their siblings). She knows the siblings maybe more than they do.

If a disabled adult is in the home, her relatives can report on her siblings, but only if they are fully knowledgeable. Otherwise, mark the disabled adult’s responses as missing.

If siblings are kidnapped or disappeared, and their status is not known please mark them “missing.”

Competent and knowledgeable adults can report for other adults in the household, but the identity of the reporter as other than the primary person must be noted on the questionnaire in Question 17. The adult must be **fully knowledgeable** about the siblings on whom he or she is reporting.

## Choosing neighborhoods and households

We are surveying 100 “clusters” across Iraq, with each cluster comprising 20 households. We will select clusters in each governorate. Fortunately, we had the assistance of a skilled person at Simon Fraser University, who developed a sophisticated cluster selection process using Google Earth maps, grids, and population data to achieve a random population proportionate-based selection of 1 square kilometer neighborhood clusters. For each cluster we have chosen a “start household” by establishing a 10 x 10 m grid on the neighborhood and picking a house randomly from this grid. This start house is noted as the Index Household (IHH) with a unique number on the maps (for example IHH_ID_FIN_16). When you look at the maps, the “start house” is indicated with a yellow pin.

The “start house” will be found on the ground by reading the maps. The supervisors will identify the start house for each cluster. In most instances this will be done a few days in advance so that the supervisor can guide the team to the start house. Once the teams go to this house, they will systematically visit the next 19 closest houses—giving a total of 20 households in each cluster. The second house will be chosen after the interview in the start house by turning your back to the main street and going to the next closest front door without crossing the street. If the next house is at the end of a blind street, cross over to the next nearest front door and proceed house-by-house to achieve the 20 households. Turn left at the end of the small street if you need more houses, unless this is impossible. In this case, turn right. In the case where the start house is on a main street, stand with you back to the door of the start house and turn left. Proceed to the next nearest front door.

Possible replacement house

**Cluster Replacement rules**

1. If there is an announcement on radio or television that there is trouble (bombing, demonstration, explosion, shooting) in an area, we will not go there until the danger is passed. Media sources include Al Jazeera, BBC, Ilhurra radio and television (Voice of America from Iraq), and the official Iraq channel, but could also include local channels or newspapers.

2. Local knowledge not announced in the media will be honored as well. If a friend or a governorate liaison calls the project manager and reports a problem in an area (demonstration, explosion, shooting), we will use that information to schedule or reschedule visits.

3. If there are guards or militias forbidding access, we will avoid the area until the blockade clears.

3. If the security problem is considered to be temporary, we will do that cluster later. The project manager will monitor the situation. We would rather do the original cluster than replace it.

4. The project manager and the two supervisors will communicate daily throughout data collection period. Supervisors will equally communicate with team members. Information can move both ways; this is a flat structure with safety and security more important than reporting protocols.

5. The project manager will keep track of all replacement decisions and the reasons for them, and will report them as part of the project reporting system.

If a **cluster** must be replaced, the project manager will pick the next neighborhood from the randomized replacement list from the same governorate. It may take 3-4 days for the Simon Fraser team to randomly pick a new start house from the neighborhood and produce new maps to find this house.

If an **Index Household or start house** must be replaced, supervisor will determine that the replacement IHH is used. For example, the first replacement choice could be labeled IHH_ID_FIN_16A. This can be avoided if there is a ‘next nearest door’ from the original index household. That will then become the new start house and the cluster will proceed from there. If this is not possible (for example the IHH 16 is not in a residential neighborhood) then the replacement IHH_ID_FIN_16A is used, or 16B or 16C.

**Household replacement rules**

1. We will not go to homes where people are hostile or threatening.

2. Households will be replaced where there is an outright refusal to participate.

3. If no one is ever home, ever after return visits, we will replace the household. If no one has returned to a vacant house by 2pm (depending on local situations) then selection of a replacement house should be considered by the supervisor.

4. We will need to replace the house if the structure originally there is completely destroyed and uninhabitable / uninhabited.

5. When a house is not occupied by a family we will replace it (for example, when the house has been converted to a business or school or similarly non-residential purpose).

6. Supervisors will keep track of all replacement decisions on the cluster cover sheet and the reasons for them.

If a household must be replaced, simply go on to the next nearest door following the usual protocol, but this must be noted after Question 7 on the form.

In each governorate, we will have someone who lives there who will work with us to help find our clusters and identify any hazards. This person will help us enter each cluster in a way that is respectful and appropriate. In some places, there may also be people from whom we need to seek permission before we can knock on doors.

*Physical safety.*  In case of physical danger, two options exist. You could come back at another time, if this is a temporary insecurity. If the danger is likely to exist for some time, then it will be necessary to select another cluster. The team must always be aware of its risks, and if there is a sudden change, the team should seek safety. Keeping regular communication with the team managers is important for the safety of all. Supervisors and field managers will work with the Technical Manager to select a replacement cluster should that be necessary.

## How to approach a household

Each household must always be approached with respect and with caution. Our goal is to obtain a completed questionnaire from each adult member of the household (*whether they are home at the time of the visit or not, even if you must come back or telephone them elsewhere*). You must also be respectful of people’s time, and the safety of the interviewers.

The purpose of the survey and the confidentiality of participants should be explained to the head of the household, or the senior person present, and any questions encouraged and honestly answered. From the beginning, you must use a scientific approach that will avoid being influenced by any of the personal views that you may have.

There is a short introductory script that asks the household head or senior member to participate in the study, and you will need to memorize this or have on hand to read. It is at the end of the manual beginning on page 27. If the household representative indicates an interest in participating, then read the consent form. The person must say verbally that he or she understands and consents to participate. If the Head of Household and others consent to participate, but one person does not, then please list that person as part of the household, but note on the household listing line for that person that they refuse to participate.

Ideally, all adult members of the household should be on hand when the consent form is read, so they will be aware of what is being consented. In any case, each adult must be asked to participate and “consented” (that is the process where you read the consent form and get verbal agreement to proceed). Before you start the individual interview please check to be sure they have consented to participate. Each adult must be given the opportunity to decline to participate or to discontinue the interview if they wish. If any adults are not present when the consent form is initially read, it should be read separately to them and their willingness to participate noted.

## Who to interview?

- If the head of household is not present, ask to see the senior adult present, that is the person most knowledgeable about the household. Either the head of household or the senior adult would be considered the **Key Informant**, and their name entered on line 1 in question 7.
- If there is one eligible respondent at home, but others are missing, see if you can collect information about the missing individuals by having the at-home person call the missing person on a cell phone. Perhaps between the at-home and missing person on the phone you can collect the necessary information. If not then you will have to make a **Call-Back** when the missing persons are present. Try to schedule this so you are sure to find this person when you come back.
- If eligible respondents are not home, and cannot be reached on the phone, you must return at least once more that day. Try to make at least 3 attempts. The quality of the survey will be diminished if we miss persons.
- There are five reasons a household could be skipped: 1) People are hostile or threatening; 2. There is an outright refusal to participate; 3. No one is ever home, ever after return visits; 4. House completely destroyed; 5. House not occupied by a family (instead has been converted to a business or school or other non-residential purpose). NOTE: Supervisors will keep track of all replacement decisions on the cluster cover sheet and the reasons for them.
- If no one is found at an empty house after three attempts, a replacement household will be chosen by the supervisor. The fact that this is a replacement interview must be noted on the household cover sheet.
- If NO ONE is home, or no eligible respondents are at home, note this on the Cluster Checklist and move to the next eligible house. Return to this home AT LEAST one more time that day, to find ALL eligible respondents to interview.

## Questionnaire script

Each questionnaire is written in Arabic and the questions translated into Kurdish where required. For many questions, it is very important to use the **exact** wording specified, to ensure that the meaning of the questions are conveyed consistently to all respondents. This is especially important for questions such as “Did you have any other brothers born of the same mother but who died young and who you forgot to name above?” Where you are to **use the specific words, this will be written in bold faced type.**

| **EVENTS CALENDAR** | |
| --- | --- |
| **YEAR** | **EVENT** |
| 1980 | Iran-Iraq War |
| 1988 | End of Iran-Iraq war |
| 1990 | Iraq invades Kuwait |
| 1991, 17 Jan | US begins Desert Storm attack on Iraq |
| 1992, Aug | No-Fly zone Northern iraq |
| 1994 | Hussein Kamil assassination |
| 2003, March, | US invades Iraq |
| 2004, Nov | Destruction of Falluja |
| 2005, Jan | Election Transitional National Assembly |
| 2006, 22 Feb | Bombing of al-Askari Mosque in Samarra |
| 2006, 30 Dec | Saddam Hussein executed |
| 2007, Dec | Turkish attacks on Kurds in Iraq |
| 2008, March | Iranian president Ahmadinejad visits |
| 2009, Feb | Parliamentary elections |
| 2010, March | Election |

For some questions the respondent may be unsure of the answer, and may need further explanation from you. This is expected. You may need to repeat the question again. If the respondent still does not understand, you may need to ***explain*** the question beyond what is just written. However, please do this as little as possible because too much explanation can change the meaning of the question. For some questions, the person may give an answer that you may have to interpret to select the best answer among the codes. This would include causes of death. The person answering the question may say her father died from chest problems, and you would have to ask a bit more to decide if this was cardiovascular disease or lung disease.

Sometimes the respondent is uncertain about the correct response and you may have to ***probe*** to help them think about the correct answer. This could be about the year a sibling was born or the cause of death of a sibling. This is common with dates. To help with the dates, use the ***Events Calendar*** . With this, the interviewer can help the respondent pinpoint the date using important or major events such well-known newsworthy events. Each interviewer will also have a card to help them convert the age in years to the year of birth.

## Understanding the questionnaires (question by question review)

### Questionnaire

The questionnaire has five sections

1. Information box
2. Consent form
3. Household listings
4. Demographic household events (births and deaths)
5. Sibling questionnaire

*1. Information box.* The box in the upper right corner lists the identity of the cluster, the interviewers and the supervisor, and whether this house is a replacement for another house in the cluster which was not included for some reason.

*2. Consent form* is the process of getting approval for the research. This process must be completed before any data collection can begin. This section is just a reminder that this must be done before any interviewing can be done.

*3. Household listing*. This form records the details of the number of persons living in the household. As a reminder, a household is a group of people who usually sleep and eat together, with a separate entrance from the outside and who share a kitchen. Members of a household may or may not be related as family. Adults and children will be listed separately on the first page in questions 7 and 8.

***Q7.*** There are spaces for 8 adults, aged 18 and older. You will list the first names or roles of all adults, their ages, their sex, whether they are present here now for the interview, if you will need to return for a call-back session or cell phone interview, and finally a column to mark when all the sibling questionnaires have been completed. This is the verification that the interview work for this household has been completed. Be sure this completion has been noted, as this is very important for the records. The first entry should be the person providing the information about the household who is the **Key Informant** for this household. Usually this will be the head of household or the senior adult in the household. If it is the senior adult, then be sure to list the Head of Household as number 2 in the list. Tick the box for the first or second line, which ever lists the head of household .

In column (b) you will list the age at last birthday, and their sex in column (c). In column (d) you will write YES or NO if they are in the household at the present time, and make a note for yourself on the next column if you will have to call back or use a telephone interview. In column (e) please enter YES if they are a sibling of someone else in the household so they will not need to be interviewed, or NO if they are not a sibling—and will be interviewed about their own siblings. In the final column (f) note when the sibling interview is completed. If there is a second or third sibling that you did not interview, please write NO in column (f).

***Box (beside Q8).*** Here you will enter the total number of all the adults in the household who you will need to interview for the sibling interview. Remember that if there are two or more siblings IN THE HOUSEHOLD, you will only interview one of them. The one to choose is the one with the birthday closest to the date of the interview. So just include the number that you will be interviewing in the number you write in the box.

***Q8.*** This lists the children in this household aged under age 18. For this question, you will list only the age of the child and their sex. For age, list only age in completed years—and for children over age one you need not complete the “months” column. The “months” column is used *only* if the child is less than one year. Use completed months for children less than one of age. For example, a two-week old infant is ‘0’ months of age.

Count up the total number of persons listed as living in the household and put the total number in the space below Q8, and ask the following question, “**Is this correct that there are XX persons living in this household (adults & children)?”** If the key informant says “No,” identify the reason for the discrepancy and go back to adjust the household listing as needed.

*4. Demographic Household events,* Births and Deaths

***Q9. The Household survey.*** This question is asked from the head of household or the senior member of the household, or the person who has been a member of the household the longest.

First it must be decided when the household began and this entered into Q.9 It could be from the time of marriage of the oldest husband and wife pair in the household. If there were an older woman living in the household who was mother of the husband or wife, then the date the household began would start with her marriage. However you are only interested in information from January 2001 onward, regardless when the household was formed. In some cases the person you are interviewing about the birth and death events in the household has joined the household sometime after it was formed. Perhaps husband and wife got married in 2004, but the mother was married in 1970. You could collect the information on births and deaths from her starting in 2001, as we don’t need any information before that date.

***Q10.*** This is for births in the household, since 2001, or when the household began. For events in 2003 be sure you note whether they occurred ***before*** or ***after*** March 2003. For each box in the table write down how many births occurred under the appropriate year. For years in which there were no births—write “0.” For years before the household began or before the person was part of the household mark a “—“. The reason for this distinction is that we want to be sure you didn’t just somehow miss filling out that year in the the questionnaire.

***Q11.*** This question asks how many different cities or districts in which the person answering the question—the head of household or the senior member of the household--- has lived since January 2001. This means the different cities, and does not include moving from one house to another within the same city. It could also include cities outside of Iraq. This applies just to the person being interviewed and not the entire current household or previous households—just where this person has lived. This is a measure of migration history.

***Q12.*** Deaths in the household since 2001. For this table you enter each death (column 1) on a different line and put a mark in the year in which it occurred. For years in which there were no deaths—write “0.” For years before the household began or before the person was part of the household mark a “—“ to indicate that the person answering the question does not have information on that year.

***Q13.*** In this section you need to provide the details on the deaths you recorded in Q12 using the same number of deaths you counted in that question. For each death you will fill out a separate line (a). You will need record the sex (b), the date of death in giving both the month and year (c). If the month cannot be recalled even with probing then enter a dash and then the year ---/correct year. (if not sure about the year, please record your best estimate) In column (d) you will write the cause of death using the codes at the bottom of the page. Please note that there are separate codes for persons under age 18 and those 18 and above. You will need to determine the age at death in order to choose the proper code. The cause that people tell you many have to be interpreted using your medical judgment to be the one that **best** describes why the person died.

The column (e) asks if you can see a death certificate for the death reported. Please politely ask for this. If you see it write 1. If they are sure it is at home, but cannot access it, then note that as (2), or record if they do not have it (3).

For column (f) you will ask whether the death was war related (yes, no, or don’t know-DK). Here “war related” means *any* conflict related events including action by the police or security forces or militia. If the death was not war related, then you are finished with that death and can go on to the next death, or the next form. If a death was war related, in column (g) please enter the code indicating “injury( war),” and in column (h) who the person you are talking with thinks was responsible.

In column (i) record whether the death was at or near home (defined as within 1 km of home), or if the death took place more than one km from home. This means where the *event* causing death occurred, such as a shooting or bomb, and not the hospital where the injured person may have died.

*5. Sibling questionnaire.* This is where you record information about all of the brothers and sisters of each adult in the household (page 3 -5 ). The person you are interviewing is not counted as a sister or brother.

***Q14-15.*** Information box: there is a box in the upper right corner. Please fill this out completely for each person you talk with. This is VERY IMPORTANT. This information is the link to the household, and is the only information that will link this page to the information in the first part of the questionnaire. Fill this information out completely before starting on the sibling history. Also note whether this interview was conducted in person or by phone. You will need a separate questionnaire sheet **for each adult** listed in question 7.

The first page of this Sibling Survey form is used to find out exactly the number of brothers and sissters what you need to find out information about. There is often confusion at first when you interview people about brothers and sisters concerning exactly who could be included. These questions help make that clear so you can go on to ask about the right people and not add or leave out any by accident

***Q16.*** Here you need to indicate which household member is reporting about his or her brothers and sisters. The individual’s number comes from the Q7, the Household Listing on the page1—a1-a8.

***Q17.*** In most cases you will be collecting information from the persons named in Q7 about their own brothers and sisters. In this case check the first box “the household member themselves (same as Q16).” However, there are some times when the best information about brothers and sisters of an adult member of the households comes from another household member and not the adult member themselves. This could be an elderly adult who does not remember his siblings well, but his daughter does. It could also be the mother or father of an adult who is currently not in the home or unable to answer for themselves. In this case, indicate who is providing the information about the adult household member listed in the space in *Q17*. One must be very careful recording information about the brothers and sisters of someone else. A mother may know about the brothers and sisters of her daughter. But a daughter may have troubles recalling births and deaths of her mother’s siblings. If there is any doubt about the ability of someone to recall about brothers and sisters of someone else, avoid interviewing that person.

**Asking about sisters**

***Q18.***  This question has multiple prompts to help the person being interviewed get to the correct answer. The first is *“How many sisters do you have?”*. This question is followed by “*How many of these sisters were born to the same mother as you were?* The purpose of this is to drop out sisters born to the same father but not the same mother (half sisters) or others who are not born to the same mother (for example, step-sisters, adopted brothers, and so on).

The next prompt question is *“Do you have any other sisters who were born of the same mother but who died young who you forgot to name?”* With this information you may change the number of sisters further by helping the person answering to remember other sisters who may have died before they were born. This includes those live births that did not live more than a few days, but it excludes stillbirths.

The final prompt question is “*Do you have any other sisters born of the same mother that you have not seen for a long time, or who you do not know are dead or alive, or for other reason who you may have forgotten to include?* This is a final reminder, but also includes the question about people who may be missing, and the household does not know where they are. Some of these persons may have been missing for many years, yet families still do not consider them dead.

At the end of this series of questions you will arrive at a final number of “true” sisters to be included in the survey and enter these into the (18) 🞏. Once you have this number, enter the names on the next page under question 20, on the next page and then come back to do the questions about the person’s brothers, write this number into the (19)🞏 .

The last question in this section is a check on the numbers. Please ask the adult you are interviewing “Now, from what you have told me, this means that the total number of brothers and sister you have is XXs. (*Q18+Q19*). Is this correct?” This is a final check to be sure this is the right number you have collected. **Do not forget to do this!** If this is not right, stop to correct this before going on.

***Q20***. For this table, enter the information about all the sisters the person being interviewed has named. In column (b) list the year of birth for brothers and sisters. If they have trouble with the year of birth, ask how much older or younger the sibling is than the person you are asking. In some cases of older siblings people may have difficulty recalling the month of birth or month of death. Please make the best estimate possible comparing with the year of birth of the person interviewed. If the month is not known after probing, enter ----/ correct year (whatever this is). It is better to have an estimate of the year than to leave it blank. In column (c) note if they are alive (Yes, or No, don’t know-DK, or Missing). If they are alive then go to sister number 2 and so on until information for all the sisters is completed.

If a person is dead or missing, please note the date the death or disappearance occurred in column (d). The location of death is in column (e) and the cause that **best** explains the death in (f). For causes of death, note the cause from the codes at the bottom of the page depending on whether the death was a child or an adult. (In the case of *Missing* or *Disappeared*, the answer would be 10 or 21-both = Don’t Know). If the death was war-related (yes or no). Here war-related means anything associated with armed violence or conflict, and would include actions taken by army, police, security forces or militia. As an example, if someone with a health condition that could have been successfully treated, but the security forces would not allow the person to go to the hospital, that is considered war-related. If the death was war related, then use the codes (h) to classify the war-related violent death. Please treat the feelings of the person you are talking to with great respect, as deaths and disappearances is a very sensitive subject, and you must be very careful with the emotions of the persons you are interviewing.

The final column (i) is to record the group or parties who the person being interviewed believes was responsible for the death of their sibling. Ask this question without suggesting any possible answers, and make your selection of the code closest to what they say. This only represents who the person *believes* might have caused the death. Many times they will not be sure, or may not want to say. Please respect this, and do not try to “put words in their mouth.” It is possible they do not want to say who they believe is responsible even if they are quite sure. This is likely to be a very emotional topic, so please ask these questions carefully and with respect to the person’s feelings.

***Q21.*** This asks the same information about all brothers of the person interviewed. The questions are the same as for the sisters. We ask for brothers and sisters separately because it helps people to count more accurately.

**End of the Questionnaire**

You have now finished and are ready to complete the questionnaire or the next adult member of the household. These questions may raise many sad and angry emotions. Please be sympathetic and treat the persons you interview with great respect and kindness. We are very grateful to the persons participating in this survey for sharing information about many deep and emotional events in their lives.

## Entry of data

At the end of each day, interviewers will forward their forms from the day to the person responsible for data entry which will be completed with a program called EpiData. The data entry program is set up to have “skip patterns” that will allow the person doing the data entry to navigate the pages more efficiently.

Treat these computers with care. Interviewers may keep their machines at the close of the survey.

Please keep the paper forms safely stored in a separate folder for each Cluster. Turn in the folder with all the household sheets to the project supervisor, who will arrange to have the data entered again for quality purposes. Make sure all pages for the same household are clipped together and labeled so they can be matched.

The project manager will save all the paper forms to bring to the final meeting with the research team.

The questions on the computer screens will be in English, although the paper survey forms will be in Arabic, so keep track of the numbers on the screen carefully. When the data is entered the same questions from your paper forms will appear on the screen. Some will be multiple choice, and sometimes you will enter short text using the keyboard. The computer program will limit the answers that can be entered to the correct range, and not allow missing data. There will be “drop-down” menus so you will not have to type in items such as governorates. The drop down menus are bought down with <F9> and are indicated on the screen by a *. The names from the paper forms are not entered into the computer database.

Once you’ve entered information, please cross people’s names off on the paper form so they can’t be read.

At the end of each day you will copy the day’s work over to a flash drive (the rec. file). The supervisor will upload data to the server on Wednesdays and Saturdays, or as soon as possible.

## Qualitative data

As you leave each household, please take a few minutes to use your digital recorder to make notes on any stories you heard or observations you made that might be of use to the researchers. Be sure to describe the reports of how any destroyed homes came to be that way if neighbors will tell you. We hope there will be enough data from these entries to write a paper or report on the events that affected households since 2003 and how these personally affected the people that you have interviewed. Start the recording with the date and your interviewer number.

# The study team

This study is only possible because there is a well trained, experienced and highly committed group of field workers in Iraq. Their ability to reach cluster sites, and their ability to collect accurate and complete data are critically important..

The field team will have three levels: four teams of 2 **interviewers** (or surveyors) who will travel in pairs, a **supervisor** for each pair of teams, and a **project manager** who oversees the entire project. Generally two teams (four people) will cover a Cluster and try to complete it in a day.

For each cluster, the **supervisor** will duplicate the interview in at least one household. This is quality check is part of all field research, so it should not be considered as a lack of trust in the interviewers. Instead it is a verification of the accuracy of the work the field teams have carried out and a way to find opportunities to improve. Having quality checks makes the results more acceptable to readers of the results. It is very important as the results this study will have many critics.

Overall direction for the study in Iraq is the responsibility of the **Project Manager**. This person will be responsible for the scientific basis of the study. The roles and responsibilities of the field teams are set out in the next section. The privacy of all persons involved in the fieldwork (both data collectors and respondents) will be fully respected in all references in the publishing of results.

Within Iraq there will be a data manager who will be responsible for entering the data from the questionnaire as soon as possible after collection as possible and for transmitting this on to data website during the same week

Outside of Iraq, there are public health scientists from three universities who have worked hard to design the scientific basis of this study, working closely with the Project Manager. Many experts from other organizations have assisted.

Once the data are collected by the field team, the university faculty in Iraq and outside will begin the analysis using multiple approaches. Once the results are completed, the team outside Iraq will consult closely with the Iraq team to help shape the presentation of the findings and be sure that details of the data collection process are accurately presented.

## Roles and responsibilities within the field team

### Project Manager:

This person has oversight of all technical matters in the field about the study. This includes location of the clusters, selection and replacement of clusters as needed, resolving uncertainty about phrasing or terminology, and making arrangements with the Ministries and other authorities. This person is the Iraq member on the scientific team responsible for the scientific integrity of the study. The Project Manager will maintain close communications with the study team at the universities.

The Project Manager will have overall responsibility for direction of field activities and systematic implementation of the survey in accordance with the procedures outlined in this field manual. The project manager will resolve any uncertainties with the supervisors, so close communication is important. The project manager will closely follow the work of the team supervisors and the team members on a daily basis either in person or by telephone. The project manager will assign a unique code number for each interviewer. The project manager will monitor the security situation closely and maintain close communications with the teams and team supervisors at all times.

*Supervisors:*

The supervisors will be responsible for locating the start house for the cluster and identifying the houses to be part of the cluster. Where this is difficult, they may employ “scouts’ to work in advance to identify the house but also the most efficient way of reaching the house, considering the many road blocks in some locations. They will make the necessary contacts and responses to the community about the nature of the survey. They will make all arrangements with the driver for transportation, and will ensure safe and secure overnight accommodation when necessary. The safety of the survey team will be the supervisor’s *immediate* responsibility. The supervisor will continuously check the work of team members, ensuring they are interviewing the correct houses and that all eligible persons in the households within the cluster have been included before moving on to the next clusters.

The supervisor will coordinate the call-backs the same day for missing members and assist in discussions with household members where there are any difficulties or misunderstandings. As necessary the supervisor will also conduct interviews. The supervisor will be responsible for reviewing all the data entered each day by the field interviewers, and ensuring that these are complete. This person is also responsible for daily movement of data forms to the data manager for entry.

For each household there will be a paper **survey form** with identification numbers of the cluster and interviewer in the corner.

For each cluster, there is a paper **Cluster Summary sheet** that must be completed at the conclusion of that cluster’s interviews (see below). On this form we will document the number of refusals for interviews, and the number of houses where no one eligible was available to interview. Any time we need to replace a household (for reasons stated above), we will document this on the Cluster Summary sheet. The Cluster summary sheet will be the primary method for tracking call backs to ensure that all eligible households have been included. They will be very important during the analysis phase of the study.

An additional function of the supervisor is to conduct a duplicate set of interviews in one household per cluster. The household selected for the duplicate interview must be selected at random, either by choosing a number between 1 and 20 from a container (“hat”) or by using the numbers on a currency bill to indicate which house to select.

| For purposes of record-keeping, the supervisor marks the name of the street on the aerial Cluster map and maps the location of the houses interviewed, with landmarks of the neighborhood. This will include an “X” for houses where no one was home or where participation was refused. This map with the locations of the households interviewed must be treated as a **confidential document.** This means it must be locked up or kept in a secure location throughout the study time. It also must be kept as a permanent part of the study records. This must be treated carefully.**Cluster cover sheet** | | |
| --- | --- | --- |
| Number of the cluster ____________________ | Date(s) cluster done __________________ | |
| Was this the originally scheduled cluster? YES ⬜ NO ⬜ | | |
| If NO—what cluster did this replace? _______________________  Why was it necessary to replace the former Cluster?Reasons include: Not residential neighborhood? Y/N  Insecure? Y/N  Notes: | | |
| Interviewers (code numbers) ____________________ | | |
| Supervisor (code number) ____________ | | |
| Duplicate household interview conducted for quality assurance: Number of house: | | |
| Total number of households interviewed | | |
| Households with no eligible person present after 3 tries | | None ⬜ or number ________ |
| People are hostile or threatening | | None ⬜ or number ________ |
| House completely destroyed | | None ⬜ or number ________ |
| House not occupied by a family | | None ⬜ or number ________ |
| Households refusing any interviews | | None ⬜ or number ________ |
| Total number replacement households required | | None ⬜ or number ________ |
| Number of houses requiring more than one visit | | None ⬜ or number ________ |
| Number of siblings living in other households in the cluster.  Note below the house number and household listing number of the individuals who are siblings of respondents in other households | | None ⬜ or number ________ |
| **NOTES** | | |

## Interviewer

The interviewers have the most important jobs. There will be 8 interviewers (four teams of two), and some substitutes. Since two teams will work together to cover a Cluster in a day, teams could be in one or two governorates at a time.

Interviewers are responsible for careful, accurate and thorough collection of data. Their priority is to ensure that *all adults in the household* are interviewed, either on the initial visit, during a call-back visit, or by cell phone if there are no other alternatives. Interviewers must work to preserve the confidentiality of household information and personal safety. Missing data are a **serious problem** for the study, so the conscientiousness of interviewers is of the utmost importance.

At the conclusion of each household interview, the interviewers and their supervisors will review the front sheet of the survey form to ensure that all activities for that household are complete or note which follow-up activities are required to achieve completion. This includes the scheduling of call back visits. The household members missing from the interviews must be noted on this form, as it is the main record for making call back visits. When the call-back visits are completed, these are crossed off the listing. Those completed by telephone are noted.

There will be five pages of data collected from each household: 1) household listing page; 2) household births and deaths form; 3) sister tally sheet; 4) brother tally sheet and 5) sibling status sheet. Each additional adult sibling generates another three sheets: 3) sister tally; 4) brother tally and 5) sibling status. Try to keep all these pages for the household together and labeled for matching.

.

We are providing digital hand-held voice recorders to each supervisor. We would like you to speak a few words into the recorder following your household interviews where there are events of interest. This is you recording, and not interviewing household members. Tell the recorder any stories you hear that are interesting or might be of use to the researchers, note any peculiarities about the household that may affect the research, and the like. Note the cluster number, but no other identifiers when you make this recording.

## Quality Checks

In each cluster, the team supervisor will check each cluster by selecting a house at random to check. S/he will put the house numbers in a container or pull out a monetary bill and choose the last two serial numbers that fall between 1 and 20, and survey that house. The team leaders will check:

1) Did the team come?

2) Count the number of adults and children in the household (page 1)

2) Count birth and death totals (page 2), but without details

3) Count siblings of all adults

5) Establish disposition of all siblings (page 4).

We will provide a separate quality check form.

Data Manager

The role of the data manager will be to

1. Receive the completed forms from the interviewers within 24 hours of interviews. Ideally the data manager will work with the teams in the field sites. The forms will be reviewed for completeness and the interviewers will be asked to correct any missing values prior to entry.
2. Data will be computer entered and checked against the paper copies for completeness.
3. When the data entry is complete for a cluster these will be transmitted to the University of Washington website or sent to the data base manager there by email attachment.
4. An update on data received and data outstanding will be maintained by the University of Washington in conjunction with the Iraq Data Manager.

# Communications

Regular and free communications among the team, both within Iraq and outside Iraq is critical to the success of the study. At the field sites (clusters) while the interviews are taking place, almost anything could happen that other interviewers urgently need to know. If something happens that your supervisor or another team needs to know about, MAKE A TELEPHONE CALL immediately. This could be something about team safety and security, or it could be about unusual findings that could affect the information being collected by other interviewers. If the issue is a safety matter that could put people interviewed or the interviewers at risk, an immediate STOP WORK ORDER would be necessary. The Project Manager will be the point persons for communications in Iraq.

# Record Keeping

## The consent form

The consent information statement is recited at the start of all interviews. No data are to be collected without consent of each respondent. This is verbal consent form. That means that after the consent form is read, and the consent is given, a signature is not required. However, the person doing the interview must note that consent has been given. If the head of household or senior members consents, then others in the household will be asked for their consent at the same time. Even if a person consents at the initial reading of the consent form to household members, that persons should be asked again to confirm permission before the interview. Every person has the right not to participate or do discontinue the interview at anytime during the interview without any consequence whatsoever. At the same time, refusals to participate in the study will affect the scientific integrity of the findings. As an interviewer, your ability to establish rapport with each subject will play a critical role in his or her decision to participate in the interview.

## Maintaining confidentiality

Privacy and confidentiality of the information the participants provide is of the greatest importance. This starts with collection of information, which must be done in a way that is private, and not overheard by others. Outside the household, the information the respondent tells you may cause some risk to them if it is known by some outside people or groups. Consider the location for the collection of information within the household. The respondent must feel at ease and comfortable to tell you all the information you are asking. Otherwise, they may withhold key information. You must tell them that after you collect information you will keep it safely. This means kept in a way that no one will be able to identify the information as belonging to them or to this particular household. Tell them that names collected will be destroyed after all of the interviews in the cluster have been completed and will not be kept permanently. The respondent must trust you and in answering the questions you are asking.

Confidentiality also means that the information you have already collected must be kept confidential.

## Security

Security means physical safety for the participants and the interviewers. A household should never be interviewed if it is likely that doing this will cause those living there to be at immediate physical risk. If you sense immediate danger, then come back later or, if the risk is anticipated to last more than a day or two, then select another site.

Interviewer safety is equally important. Use local information from those familiar with the governorate and local area. Use your personal sense about what is dangerous. If dangers develop during the surveys, then the right thing to do is leave. It is important that your presence does not arouse suspicion or create misconceptions as to why you are present. The team (including the driver) must be ready with an explanation of why you are present in the community. Clearly explain the nature of your survey to all who enquire. Your profession as health workers will provide you some degree of protection, and do not hesitate to use this information. If necessary, show a copy of the Ministry of Health letter of permission for the survey. Where possible, a local health worker will be added as part of the team. This person will have the local references and contacts that will add safety and local information for the team.

Communications are an important part of risk reduction. Regular telephone conversation with the project manager and other team members by telephone is part of a communications plan that will reduce risk to the team members. Sharing experiences and coping methods can help reduce risks.

### Safety rules:

#### Confidentiality

1. Names of the team members are private.

2. Do not disclose involvement of Americans or Canadians in the project.

3. Movements of the team will be undisclosed until only one day in advance for the Baghdad area. For movement to out of town locations, we will say how long they will be gone, but won’t tell them where they are going until we are underway.

4. Team members are to keep a low profile, not appear in the media.

#### Documents

1. Papers will be in Arabic, not English.

2. Team members should carry approval letters.

3. Keep maps out of sight and don’t flash them around in the street.

#### Phone numbers

1. Everyone will have mobile numbers of all team members.

2. Supervisors will have family contact information for all team members.

#### Trust

1. The project manager will hire only people who are well known to him or the supervisors.

2. Coordinate with the local governorate liaison.

#### Tracking

1. The project manager will follow the movement of the team the whole way there, during and back.

#### Driver and movement

1. Driver will be unarmed, but will serve as a guard to the team.

2. Driver will have a cell phone and communicate with team on the ground.

3. If the car breaks down, the driver will call the project manager.

4. Cars should travel with water, food reserve, first aid kit, spare tire, tools.

## Household recruitment script (see Arabic translation)

**­­­IRAQ POPULATION DEMOGRAPHIC SURVEY 2011**

**Household recruitment script.**

Hello, my name is___________________.

I am with Mustansiriya University Medical College, and we are conducting a research study seeking to document national demographic changes in Iraq over the last ten years. To do this, we are asking people in your neighborhood, and in other neighborhoods in Iraq chosen randomly, to tell us about their children, their siblings and their household members—-when they were born, whether they are alive or dead, when they might have died, and of what cause. Respondent participation is entirely voluntary and you may discontinue participation at any time without any consequence.

Are you interested in hearing more about this study?

(if yes, proceed) (if no, thank you, and sorry to disturb)

Is this a good time?

(if yes, proceed) (if not, when may I return?)

May we please speak with the head of household, or the person here now who is most senior? We would like to present the information about our study to that person.

(Surveyor: Wait for senior person to be presented.)

Thank you for agreeing to consider participating in our study. We would like to interview every adult member of your household. We will ask some questions about all your ADULT household members and all of their siblings—-when they were born, whether they are alive or dead, when & where they might have died, and of what cause.

You can decide if you want to be in the study or not. As I said before, you can stop at any time and you can decline to answer any of our questions. We will not ask you to sign anything. We will give you information about the study and who to contact if you have questions for your records.

We will collect only first names or initials, and we will retain those names for ONLY A SHORT TIME. When you are done telling us about these family members, we will cross off the information so it can’t be read. No individual information will be reported from this study.

We have a supervisor who will re-check one house in this neighborhood when we are done today, to make sure we did a good job. If s/he chooses this house, s/he will ask you about our interview today.

If you would like more information about the study, or information about your rights as a respondent, you may contact the number of the study manager at the bottom of this information sheet that I am SHOWING you now. (Present information sheet.)

We realize that it can be distressing to discuss deaths of household or family members. If at any point it is too hard for you to discuss this, we will stop our questions. Another thing that is very unlikely, but could happen, is that our computers or records are lost or stolen and your information could be disclosed. Without your names it will be hard to identify you, but it is always possible.

It is not likely you will personally benefit from the study, but the country will benefit from knowing how death rates have changed over the last 10 years.

This survey will take between 10 and 30 minutes, depending on how many siblings you have.

Are you still interested in participating in this study?

(if yes, proceed) (if no, thank you, and sorry to disturb)

**INFORMATION STATEMENT TO BE OFFERED TO SUBJECTS**

**[­­­IRAQ POPULATION DEMOGRAPHIC SURVEY 2011]**

**24-hour emergency telephone number: 0770-9819584**

Mustansiriya University Medical College, in collaboration with a other universities, is conducting a research study to document national demographic changes in Iraq over the last ten years.

To measure demographic changes, we are asking people in your neighborhood, and in other neighborhoods in Iraq chosen randomly, to tell us about their children, their siblings and their household members—-when they were born, whether they are alive or dead, when they might have died, and of what cause. Respondent participation is entirely voluntary and you may discontinue participation at any time without any consequence.

We seek to speak with the head of household, or the person here now who is most senior to obtain permission to conduct the study. We also ask to interview every adult in the household. We have some questions about all household members since 2001 or whenever this household was formed (whichever is later) and the siblings of all adult household members—-when they were born, whether they are alive or dead, when & where they might have died, and of what cause.

Respondents can decide if you want to be in the study or not. Respondents can also refuse to answer any specific question and can stop at any time. We will not ask respondents to sign anything.

We will collect only first names or initials, for only long enough to understand the family composition. No individual information will be reported from this study.

Our supervisor may return to this household to check on our work to see if we did a good job.

If you would like more information about the study, or information about your rights as a respondent, you may call the number at the bottom of this information sheet.

It is not likely you will personally benefit from the study, but the country will benefit from knowing how birth and death rates have changed over the last 10 years.

This survey takes between 10 and 30 minutes, depending on how many siblings you have.

We realize it can be distressing to discuss deaths of household or family members. If at any point it is too hard for you to discuss this, we will stop our questions. Another thing that is very unlikely, but could happen, is that our computers or records are lost or stolen and your information could be disclosed.

**Contact Information**

Should you have any questions about this research at any time, please contact

0770-9819584.
