# Supplementary material for: Mortality in Iraq Associated with the 2003–2011 War and Occupation: Findings from a National Cluster Sample Survey by the University Collaborative Iraq Mortality Study
Source: PLoS Med. 2013 Oct 15;10(10):e1001533. doi: 10.1371/journal.pmed.1001533 (PMC3797136; doi:10.1371/journal.pmed.1001533)
Supplement: Questionnaire S1 — Questionnaire used by data collection personnel. (DOCX) [file pmed.1001533.s003.docx]

**S1- IRAQ POPULATION DEMOGRAPHIC**

1. Cluster No._______ 2. HH No._____

3. Governorate _______________

4. Day _____ of Month____________

5. Interviewers _______________

6. Supervisor ______

🞏 household is a replacement for another

**SURVEY 2011**

***Read introductory script, consent form*** *and show permissions.)* Please read all text in **Bold type.**

**7. Would you please list all the adults (over 18 years) who live in this household, and their age on their last birthday, including yourself?** *Start with the Key Informant—the person who knows the most about the household. Note: a household is a group of people who usually sleep and eat together, with a separate entrance and a common kitchen.*

| No.  (a) | **ADULTS 18 and**  **older**  (first names only or sibling number)  *X if Household head* | **Age at last birthday**  (b) | **Sex**  **M/F**  (c) | Present here now for interview? *Yes/No*  (d) | Need to return?  or call on cell phone to reach? | Sibling in HH?  indicate which ones  *Yes/No*  (e) | Sibling  Survey done  *Yes/No*  (f) |
| --- | --- | --- | --- | --- | --- | --- | --- |
| **1** Key Informant for HH info | ⬜ HH head? |  |  |  |  |  |  |
| **2** | ⬜ HH head? |  |  |  |  |  |  |
| **3** |  |  |  |  |  |  |  |
| **4** |  |  |  |  |  |  |  |
| **5** |  |  |  |  |  |  |  |
| **6** |  |  |  |  |  |  |  |
| **7** |  |  |  |  |  |  |  |
| **8** |  |  |  |  |  |  |  |
| *NOTE: YOU WILL NEED TO INTERVIEW all the above to get their sibling histories—UNLESS there are siblings of another person listed for this Household. If there are adult siblings living together, only one of the siblings needs to be interviewed. Chose the one with the birthday closet to today’s date.* | | | | | | | |

| No.  (a) | **Children under 18**  **(first names or initials only or sibling number)** | **Age (years) Months only if under 1 year**  (b) (c) | | **Sex**  **M or F**  (d) |
| --- | --- | --- | --- | --- |
| **1** |  | Years | months |  |
| **2** |  |  |  |  |
| **3** |  |  |  |  |
| **4** |  |  |  |  |
| **5** |  |  |  |  |
| **6** |  |  |  |  |
| **7** |  |  |  |  |
| **8** |  |  |  |  |

**8. Now would you please list all the children who live in this household (under age 18). at last birthday?**

How many sibling interviews will you need to complete?

⬜

(g)

**🡺Is this correct that there are ____ persons living in this household (adults + children)?**

**9. When was this household formed?** *(explain as needed)*

(Note: a household is formed when people start living together, regardless of the location. This usually happens with a marriage, or when adults leave their family of origin and form their own home. (A HOUSE isn’t the same as a HOUSEHOLD). Please ask the oldest person in the household.

**10. Since you joined the household (**if that was before 2001, start with January 1, 2001**) how many births have there been to all persons living in this household?** This information from the Key Informant listed as number 1 in question 7.

*(List number of births for each year). Put a” –“ for each year you were not in the household and a “0” for the years you were in the household and there were no births.*

| 2001 | 2002 | 2003  before invasion | 2003  after invasion | 2004 | 2005 | 2006 | 2007 | 2008 | 2009 | 2010 | 2011 |
| --- | --- | --- | --- | --- | --- | --- | --- | --- | --- | --- | --- |
|  |  |  |  |  |  |  |  |  |  |  |  |

**11. How many times have you moved to a different city or district since Jan 2001?  *0 if none***

*This information from person listed as number 1 in question 7.*

**12. Since you joined the household, have there been any deaths in this household among persons who were regularly living in this household?**  *Put ” –“ in the box for each year you were not in the household and a “0” for the year that there were no deaths and a X in for the year of death. This information from Key informant listed as number 1 in question 7.*

| Death no. | 2001 | 2002 | 2003  before invasion | 2003  after invasion | 2004 | 2005 | 2006 | 2007 | 2008 | 2009 | 2010 | 2011 |
| --- | --- | --- | --- | --- | --- | --- | --- | --- | --- | --- | --- | --- |
| 1 |  |  |  |  |  |  |  |  |  |  |  |  |
| 2 |  |  |  |  |  |  |  |  |  |  |  |  |
| 3 |  |  |  |  |  |  |  |  |  |  |  |  |
| 4 |  |  |  |  |  |  |  |  |  |  |  |  |
| 5 |  |  |  |  |  |  |  |  |  |  |  |  |

**13. For each death noted in question 12, please provide details:** information from Key Informant. *If the remember age of death but not year use Events Calendar. For cause of death (d & g)select the choice that most closely matches the description provided by the person—Do not read out the options.*

| Death no.  (a) | **Sex**  M/F  (b) | **Date of death?**  **month & year**  (c) | **What was the cause of death?** (codes)  (d) | **May I see the death**  **certificate?**  (e) | **Was the death war related?**  Y/N/DK  (f) | *If war-related,* **What was the cause?** (codes)  (g) | *If war related,* **Who do think was responsible?** Codes)  (h) | **Was fatal event at the house or > 1km away?**  (i) |
| --- | --- | --- | --- | --- | --- | --- | --- | --- |
| 1 |  |  |  |  |  |  |  |  |
| 2 |  |  |  |  |  |  |  |  |
| 3 |  |  |  |  |  |  |  |  |
| 4 |  |  |  |  |  |  |  |  |
| 5 |  |  |  |  |  |  |  |  |

**(d) Under 18 causes of death**: **1**=diarrhea, **2**=respiratory, **3**=pre-term, **4**=neonatal causes, **5**=injury (not war),

**6**=injury (war), **7**=neonatal tetanus, **8**=cancer/tumor **9=**other, **10=**don’t know,

**(d) 18 and over Adult causes of death**: **11**=cardiovascular, **12**=cancer, **13**=injury (not war), **14**=injury (war),

**15**=lung disease, **16**=liver disease, **18**=maternal, **19**=kidney condition, **20**=other, **21**=don’t know

**(g) Causes of violent death related to war:** **1**=road accident that was war related, **2**=gunshot, **3**=car bomb, **4**=airstrike, **7**=other explosion, **8**=other war injury; **9**=don’t know

**(h) Responsible parties for war deaths:** **1**=criminals, **2**=Iraq police (security forces), **3**=Iraq army, **4**=coalition forces, **5**=militias, **6**=unknown, **7**=other

**(j) Death certificate: 1**=able to see the death certificate, **2**=told there is a death certificate, but did not see or

**3**=death certificate not available.

**IRAQ POPULATION DEMOGRAPHIC**

14. Cluster no___ HH no____

15. Interviewer no_____

15b 🞏 in person 🞏 by phone

**SURVEY- sibling history page(s) *INTERVIEWERS: please read out all questions in bold type.***

**16**. For which adult household member (from page one) which person is reporting about their brothers and sisters?

(no.) (a)_______

**17**. Who is providing the information for the brothers and sisters of the person listed in 16?

⬜ the household member themselves (same as Q16) ⬜ another HH member (list no.) _________

**Now I would like to ask you about your brothers and sisters--that is all of the children born to your natural mother, including those who are living with you, those living elsewhere, and those who have died.**

**18. Sisters: First I would like to ask you about your sisters**

*●* **How many sisters do you have?**

**● How many of these sisters were born to the same mother as you were?**

**● Do you have any other sisters who were born of the same mother but who died young who you forgot to name above?** *Exclude stillbirths*

**● Do you have any other sisters born of the same mother that you have not seen for a long time, or whom you do not know are dead or alive, or for other reason who you may have forgotten to include?**

**(18)**🞏*final number of sisters*

🡺**Now fill the names for these sisters into the first column of Sisters Section starting with the oldest (Q20)**

**19. Brothers: Now I would like to ask you about your brothers**

**● How many brothers do you have?**

**● How many of these brothers were born to the same mother as you were?**

**● Did you have any other brothers who were born of the same mother but who died young who you forgot to name above?** *Exclude stillbirths*

**● Do you have any other brothers born of the same mother who you have not seen for a long time, or whom you do not know are dead or alive or for other reasons who you may have forgotten to include?**

**(19)**🞏 *final number of brothers*

**🡺Now fill the names for these brothers into the first column of Brothers Section starting with the oldest (Q21)**

**Now from what you have told me, this means the total number of brothers and sisters you have is ________ excluding yourself? Is this correct?20. Sisters**

**Now I would like to ask you questions about each of your sisters. Remember we are talking about all girls born alive to your mother (except you), starting with the oldest.** *(read all parts in bold to the person)*

| Number **(a)** | Sister’s First name or initials | **Sister’s year of Birth** *If unsure ask* **”How many years older or younger is she than YOU?”**  **(b)** | **Is this sister alive now?**  Yes/No/DK/Missing  *if alive skip to next sister*  **(c)** | If dead or missing**, What is date of death or disappearance?**  month/year  **(d)** | **Where did she die?**  (Governorate)  **(e)** | **What was her cause of death?** (use code that best describes)  **(f)** | **Was this a war- related death?**  Y/N/DK  *If NO-go to next sister*  **(g)** | **If war-related, what caused her death?**  (use codes)  **(h)** | **If from war, who do you think was responsible?** (use codes)  **(i)** |
| --- | --- | --- | --- | --- | --- | --- | --- | --- | --- |
| 1 |  |  |  |  |  |  |  |  |  |
| 2 |  |  |  |  |  |  |  |  |  |
| 3 |  |  |  |  |  |  |  |  |  |
| 4 |  |  |  |  |  |  |  |  |  |
| 5 |  |  |  |  |  |  |  |  |  |
| 6 |  |  |  |  |  |  |  |  |  |
| 7 |  |  |  |  |  |  |  |  |  |
| 8 |  |  |  |  |  |  |  |  |  |
| 9 |  |  |  |  |  |  |  |  |  |
| 10 |  |  |  |  |  |  |  |  |  |

**(f) Under 18 causes of death**: **1**=diarrhea, **2**=respiratory, **3**=measles, **4**=neonatal causes, **5**=infection injury (not war), **6**=cancer/tumor **7**=injury (war), **8**=infection,; **9=**other; **10=**don’t know,

**(f) 18 and over Adult causes of death**: **11**=cardiovascular, **12**=cancer/tumor, **13**=injury (not war), **14**=injury (war), **15**=lung disease, **16**=liver disease, **18**=maternal causes, **19**=kidney condition, **20**=self-inflected injury, **21**=don’t know

**(h) Causes of violent death related to war:** **1**=road accident that was war related, **2**=gunshot, **3**=car bomb,

**4**=airstrike, **7**=other explosion, **8**=other war injury; **9**=don’t know

**(i) Responsible parties for war deaths:** **1**=criminals, **2**=Iraq police (security forces), **3**=Iraq army, **4**=coalition forces, **5**=militias, **6**=unknown, **7**=other

**6**=unknown, **7**=other

**21. Brothers Now I would like to ask you questions about each of your brothers. Remember we are talking about all boys born alive to your mother (except you), starting with the oldest** *(read all parts in bold type to the person)*

| Number **(a)** | Brother’s First name or initials | **Brother’s year of Birth** *If unsure ask* **“How many years older or younger is he than YOU?”**  **(b)** | **Is this brother alive now?**  Yes/No/DK/Missing  *if alive skip to next brother*  **(c)** | If dead or missing**, What is date of death or disappearance?** month/year  **(d)** | **Where did he die?**  (Governorate)  **(e)** | **What was his cause of death?** (use code that best describes)  **(f)** | **Was this a war-related death?**  Y/N/DK  *If NO-go to next brother*  **(g)** | **If war-related, what caused his death?**  (use codes)  **(h)** | **If from war, who do you think was responsible?** (use codes)  **(i)** |
| --- | --- | --- | --- | --- | --- | --- | --- | --- | --- |
| 1 |  |  |  |  |  |  |  |  |  |
| 2 |  |  |  |  |  |  |  |  |  |
| 3 |  |  |  |  |  |  |  |  |  |
| 4 |  |  |  |  |  |  |  |  |  |
| 5 |  |  |  |  |  |  |  |  |  |
| 6 |  |  |  |  |  |  |  |  |  |
| 7 |  |  |  |  |  |  |  |  |  |
| 8 |  |  |  |  |  |  |  |  |  |
| 9 |  |  |  |  |  |  |  |  |  |
| 10 |  |  |  |  |  |  |  |  |  |

**(f) Under 18 causes of death**: **1**=diarrhea, **2**=respiratory, **3**=measles, **4**=neonatal causes, **5**=infection injury (not war), **6**=cancer/tumor **7**=injury (war), **8**=infection,; **9=**other; **10=**don’t know,

**(f) 18 and over Adult causes of death**: **11**=cardiovascular, **12**=cancer/tumor, **13**=injury (not war), **14**=injury (war), **15**=lung disease, **16**=liver disease, **18**=maternal causes, **19**=kidney condition, **20**=self-inflected injury, **21**=don’t know

**(h) Causes of violent death related to war:** **1**=road accident that was war related, **2**=gunshot, **3**=car bomb,

**4**=airstrike, **7**=other explosion, **8**=other war injury; **9**=don’t know

**(i) Responsible parties for war deaths:** **1**=criminals, **2**=Iraq police (security forces), **3**=Iraq army, **4**=coalition forces, **5**=militias, **6**=unknown, **7**=other

**6**=unknown, **7**=other
