# Supplementary material for: Mortality in Iraq Associated with the 2003–2011 War and Occupation: Findings from a National Cluster Sample Survey by the University Collaborative Iraq Mortality Study
Source: PLoS Med. 2013 Oct 15;10(10):e1001533. doi: 10.1371/journal.pmed.1001533 (PMC3797136; doi:10.1371/journal.pmed.1001533)
Supplement: Text S1 — Additional methods. (DOCX) [file pmed.1001533.s004.docx]

# Text S1- Mortality in Iraq associated with the 2003-2011 war and occupation

SUPPLEMENTAL INFORMATION on METHODS

## Additional information on sample selection

The two-stage cluster sampling employed a gridded population dataset and a spatial sampling algorithm to select clusters in a geographic information system. We used the 2008 LandScan ^TM^ gridded population dataset from Oak Ridge National Laboratory,[^1^](#_ENREF_1) a geo-referenced dataset of grid cells with values representing estimated population counts at a resolution of approximately 1 km^2^, to generate a probability surface raster. This raster layer indicated the probability of selection based on the LandScan^TM^ 2008 population estimates where higher values indicated a higher probability of inclusion. Next, we employed the “create-spatially balanced points tool” (CSBP) in ArcGIS10 to randomly select an initial set of 1km^2^ clusters based on the probability surface and the Reversed Randomized Quadrant-Recursive Raster algorithm.[^2^](#_ENREF_2) We wanted 100 clusters in the study. We initially selected 125 clusters, however, so that we could have some “spares” in case any of the first 100 provided unusable. Of the 125 clusters initially selected, 12 were obviously either industrial areas, commercial areas, or otherwise not residential according to satellite imagery and thus excluded from the sample. Using the remaining 113 suitable clusters, the first 100 were selected and the remaining 13 were identified as replacement clusters, if needed. See web appendix supplemental Figure 1.

In the second stage of sampling, we selected starting dwellings in each of the 1 km^2^ grid cells that were selected in stage one in the following manner. The starting dwellings were randomly selected using a sampling grid superimposed over Google Earth^TM^ imagery.[^3^](#_ENREF_3) We generated a sampling grid at a resolution of 10 meters by 10 meters (10m x 10m) for each cluster in ArcGIS. Subsequently, these sampling grids were exported from ArcGIS as KML files and superimposed onto Google Earth TM imagery. We assigned each 10m by 10m cell within the sampling grid a unique number from 0-9999. Employing a random number generator, we chose a single grid cell in each cluster. If the chosen grid cell contained a dwelling rooftop, that was identified as the starting house. If the single grid cell did not contain a dwelling roof, a new individual grid cell was selected in the same manner until a cell containing a dwelling was selected. In the event that more than a single rooftop existed within the selected grid cell, the dwelling with a greater proportion of its rooftop contained within the cell was selected. We took the precaution of selecting three replacement starting houses for each of the 100 originally selected dwellings, in case our maps no longer reflected conditions on the ground. We produced paper maps for the survey teams, who expressed concern that using hand-held GPS units would appear suspicious to local residents.

We established a protocol for selecting 19 dwellings adjacent to the starting household in a systematic and replicable fashion. To avoid any “main street bias,” interviewers were instructed to “turn your back to the main (busy) street and go to the next closest front door without crossing the street.” Additional instructions clarified what to do in situations such as a cul de sac, insufficient number of households, and the presence of dwellings not being used as residences. If the person answering the door declined to participate, the next closest front door became the replacement dwelling. Interviewers recorded the use of any replacement dwellings. In a single case, we used a new start dwelling when the original house on the map no longer existed. When dwellings were vacant, or adult members were missing at the time of the visit, the teams returned at the end of the day for a second visit. See additional details in companion publication: ***Galway LP, Bell N, Al Shatari SA, Hagopian A, Burnham G, Flaxman A, et al. A two-stage cluster sampling method using gridded population data, a GIS, and Google EarthTM imagery in a population-based mortality survey in Iraq. International journal of health geographics. 2012;*** *11****(1): 12.***

The mother of adult siblings in the home was allowed to report about her own children. If an adult in the home was incapable of responding, his or her relatives reported on that person’s siblings, but only if they said they were fully knowledgeable. Otherwise, the person’s response was marked as missing. If siblings lived together in the same home, we interviewed only one. Where necessary, telephone interviews of absent adult household members were conducted while the interviewers were in the household.

## Additional material on recall bias assessment

Our sibling survey found adult mortality lower than expected in the pre-war period. We conducted a sensitivity analysis to assess the possible role of recall bias. Rajaratnam and colleagues estimated 45q15 for Iraqi males in 2001 to be 147.3, based on a synthesis of all previously-available data.^4^ If recall bias were completely responsible for the discrepancy between their estimate (147) and ours (106), this would imply that recall bias was responsible for a rate reduction of 3.46% per year. This is a high rate, but within the range found by Obermeyer et al, who found recall bias ranged between -0.85% and 7.8% of time period prior to survey in a synthesis of surveys from 44 countries.^5^ As a sensitivity analysis, we recalculated our estimates assuming a 3.46% reduction per year was present in our measurements because of recall bias. We found our results to be robust to potential recall bias---because recall bias affects both the pre-war and the post-invasion figures.

## Sibling Survey methods and analysis

The protocol for this survey was to interview just one sibling per sibship within each household, although the ICSS method was designed to include as respondents all eligible siblings in a household.^6,7^ To apply the method, we first expanded our sample size by duplicating sibling responses for households where the respondent was living with a sibling. We then expanded to the sibling level (i.e. one observation for each sibling as opposed to sibship), so that the number of observations listed in the dataset for each sibship corresponds to the sibship size (*S_j_*). We then calculated weights based on the number of siblings who survived and were eligible to be included in the survey for sibling *i* in sibship *j*. For example, in a case where we interviewed a brother that lived in the same household as one of two surviving sisters, we processed the data from this sibship as follows: first, we doubled the sibship observation as if we had also interviewed the co-resident sister, assuming she would have reported the same information about the same siblings. Then we expanded the two sibship-level records to the sibling level: each of them becomes three observations, one for each sibling in the sibship. Finally the weight assigned to each of these observations was 1/3, since all siblings in the sibship had survived and were eligible to be selected into the survey.

To correct for zero-reporter bias, we followed the procedure outlined by the ICSS method.[^7^](#_ENREF_9) Details of this procedure can be found in the reference, but briefly, it entails estimating directly the number of sibling deaths that were missing from the sample by age and sibship size through iteration for sibship sizes of one and two. We then added these missing siblings to the observed sample (birth dates were assigned based on the cohort for which the missing siblings were estimated, and dates of death assigned based on the observed mortality rates for the same cohort in our sample) before calculating final age-specific mortality rates.

Data about adult mortality using the sibling report method are subject to predictable biases. Sibships that experience a higher mortality risk are under-represented at the time of the survey, because these siblings are less likely to survive to be able to report (survival bias). Additionally, larger sibships are overrepresented in the sample, because there are more siblings in the sampling frame.

The ICSS method generates age-, time-, and sex-specific estimates of adult mortality rates. In applying the zero-survivor correction, 3.6 missing siblings were added to the data set.

Citations

1. LandScan Home. available at: http://www.ornl.gov/sci/landscan/. [cited; Available from:

2. Linard C, Alegana VA, Noor AM, Snow RW, AJ T. A high resolution spatial population database of Somalia for disease risk mapping. International journal of health geographics. 2010; **9**(1).

3. Google Earth. [cited; Available from: http://www.google.com/earth/index.html

4. Rajaratnam JK, Marcus JR, Levin-Rector A, Chalupka AN, Wang H, Dwyer L, et al. Worldwide mortality in men and women aged 15-59 years from 1970 to 2010: a systematic analysis. Lancet. **375**(9727): 1704-20.

5. Obermeyer Z, Rajaratnam JK, Park CH, Gakidou E, Hogan MC, Lopez AD, et al. Measuring adult mortality using sibling survival: a new analytical method and new results for 44 countries, 1974-2006. PLoS Med. 2010; **7**(4): e1000260.

6. Trussell J, Rodriguez G. A Note on the Sisterhood Estimator of Maternal Mortality. Studies in Family Planning. Nov-Dec 1990; **21**(6): 334-46.

7. Rajaratnam J, Levin-Rector A, Murray C. Improved analysis of sibling survival data taking into account survivor bias, zero-surviving reporters and recall bias. In: Murray C, Lopez A, Wang H, editors. Mortality Estimation for National Populations: Methods and Applications. Seattle: University of Washington Press; 2012.
